# Hsc70-2 is required for *Beet black scorch virus* infection through interaction with replication and capsid proteins

**DOI:** 10.1038/s41598-018-22778-9

**Published:** 2018-03-14

**Authors:** Xiaoling Wang, Xiuling Cao, Min Liu, Ruiqi Zhang, Xin Zhang, Zongyu Gao, Xiaofei Zhao, Kai Xu, Dawei Li, Yongliang Zhang

**Affiliations:** 1 State Key Laboratory of Agro-Biotechnology and Ministry of Agriculture Key Laboratory of Soil Microbiology, College of Biological Sciences, China Agricultural University, Beijing, 100193 P. R. China; 2 Jiangsu Key Laboratory for Microbes and Functional Genomics, Jiangsu Engineering and Technology Research Center for Microbiology, College of Life Sciences, Nanjing Normal University, Nanjing, 210046 P. R. China

## Abstract

Dissecting the complex molecular interplay between the host plant and invading virus improves our understanding of the mechanisms underlying viral pathogenesis. In this study, immunoprecipitation together with the mass spectrometry analysis revealed that the heat shock protein 70 (Hsp70) family homolog, Hsc70-2, was co-purified with beet black scorch virus (BBSV) replication protein p23 and coat protein (CP), respectively. Further experiments demonstrated that Hsc70-2 interacts directly with both p23 and CP, whereas there is no interaction between p23 and CP. Hsc70-2 expression is induced slightly during BBSV infection of *Nicotiana benthamiana*, and overexpression of Hsc70-2 promotes BBSV accumulation, while knockdown of Hsc70-2 in *N*. *benthamiana* leads to drastic reduction of BBSV accumulation. Infection experiments revealed that CP negatively regulates BBSV replication, which can be mitigated by overexpression of Hsc70-2. Further experiments indicate that CP impairs the interaction between Hsc70-2 and p23 in a dose-dependent manner. Altogether, we provide evidence that besides specific functions of Hsp70 family proteins in certain aspects of viral infection, they can serve as a mediator for the orchestration of virus infection by interacting with different viral components. Our results provide new insight into the role of Hsp70 family proteins in virus infection.

## Introduction

*Beet black scorch virus* (BBSV) is a positive-strand RNA virus belonging to the genus *Betanecrovirus* in the family *Tombusviridae*^1^. BBSV genome consists of a 3644 nucleotides single-stranded RNA without a 5′ cap structure nor 3′ polyA tail. The BBSV genome contains six open reading frames (ORFs). The 5′ proximal ORF encodes the auxiliary replication protein p23, which induces the rearrangement of the ER to form virus replication complexes (VRCs)^2^. The stop codon of p23 ORF is read-through to generate the RNA dependent RNA polymerase (RdRp) protein, p82. p7a, p7b, and p5′ are translated from sub-genomic RNA1 (sgRNA1) and are responsible for viral cell-to-cell movement. The CP ORF at the 3′ proximal region of the genome, is translated from sgRNA2. CP expressed from sgRNA2 is not essential for cell-to-cell movement in *Chenopodium amaranticolor*, but is indispensable for systemic infection in *Nicotiana benthamiana*^1,3,4^. In addition, BBSV virion assembly and virus systemic movement require specific CP N-terminal basic amino acid and phosphorylation of the CP^5,6^.

Replication of positive-stranded RNA virus is a complicated but well-organized process, which involve the coordinate functions of viral proteins and diverse host factors^7,8^. Because host factors involved in viral replication represent potential targets for virus control, identification and functional characterization of their roles is one of the hot topics in virus research. Both auxiliary replicase protein and RNA-dependent RNA polymerase (RdRp) are the key components of VRC and have potential to be baits for identifying host interactors. However, molecular weight of auxiliary replicase protein is smaller than that of RdRp, which makes it relatively easy to be manipulated for the subsequent biochemical analysis. More importantly, in contrast to RdRp with confined function in viral RNA synthesis, auxiliary replicase proteins, such as tomato bushy stunt virus (TBSV) p33^9^, cucumber necrosis virus (CNV) p33^10^, carnation Italian ringspot virus (CIRV) p36^11^, and red clover necrotic mosaic virus (RCNMV) p27^12^, are multifunctional proteins and play diverse roles in virus replication. Therefore, auxiliary replicase proteins are always selected as baits to identify host factors involved in viral replication^13,14^. Recently, accumulating evidences indicated that CP is also a multifunctional protein besides its main role in viral RNA encapsidation^15^. For example, Chkuaseli *et al*. reported that deletion of CP in *Tobacco necrosis virus-D* (TNV-D), also a member of the genus *Betanecrovirus*, leads to the increased viral RNA accumulation^16^, suggesting that CP may also function in modulating the viral replication. Although CP can negatively regulate viral replication by binding to the RNA elements within the 5′-UTR^17,18^, whether host factors participate in the CP-mediated suppression of viral replication remains poorly investigated.

In this study, using the p23 and CP as the bait proteins, we found that Hsc70-2 exists in both CP and p23-associated complexes. As the core components of the cellular chaperone network, the primary function of Hsp70 family proteins is the folding of newly synthesized proteins, refolding of aggregated proteins, assembly and disassembly of large macromolecular protein complexes, translocation of organellar and secretory proteins, protein degradation, and protection of the proteome from stress^19–22^. Some members of Hsp70 family are constitutively expressed and are referred to as heat shock cognate 70 kDa protein (Hsc70), but are also induced by environmental stresses^23–27^. Previous studies indicated that Hsp70 and Hsc70 may complement each other to maintain cellular integrity during metabolic challenges^28^. The Hsp70s (referring to both Hsp70 and Hsc70) possess two domains, an N-terminal ATPase domain, and a C-terminal peptide-binding domain^20^. All characterized Hsp70s act via ATP-dependent cycles of protein binding and release^29^. Despite the fundamental role in protein quality control, Hsp70s are tightly associated with multiple aspects of viral infection cycle, such as cell entry, virion assembly and disassembly, genome replication and viral gene expression^27,30–32^. For example, Hsp70 is required for both entry and post-entry steps of the dengue virus life cycle and stabilizes viral NS5 and CP^33^. A cytosolic Hsp70 is found to play multiple roles in TBSV replication, such as the insertion of the viral replication proteins into peroxisomal membranes and activation of TBSV replication by interaction with viral replication proteins^34–38^. Recent studies also indicated that Hsc70 is co-opted by CNV to functions in several aspects of the viral infection cycle, including the positive regulation of viral RNA accumulation, virus particle assembly and disassembly, and targeting of CP to the chloroplasts^27,39^. Additionally, a chloroplast Hsp70 isoform was reported to interact with bamboo mosaic virus (BaMV) replicase and is required for the preference of BaMV for older leaves of *N*. *benthamiana*^40^.

Virus replication is a complicated process, which requires temporal, spatial and mechanistic coordination of various host factors and viral proteins to ensure the efficient multiplication of virus^9,41^. Due to the association of Hsc70-2 protein with both BBSV p23 and CP, we want to investigate how the Hsc70-2 was coordinately manipulated by different viral components during viral infection cycles. Our results showed that Hsc70-2 is essential for BBSV replication through interaction with the p23, whereas CP disrupts the interaction between Hsc70-2 and p23 in a dose-dependent manner, and overexpression of Hsc70-2 mitigates the negative effect of CP on BBSV replication. These data suggest a new role of Hsc70-2 in regulating BBSV replication through interaction with different viral components.

## Results

### Hsc70-2 was co-purified with BBSV p23 and CP

**Figure 1:**
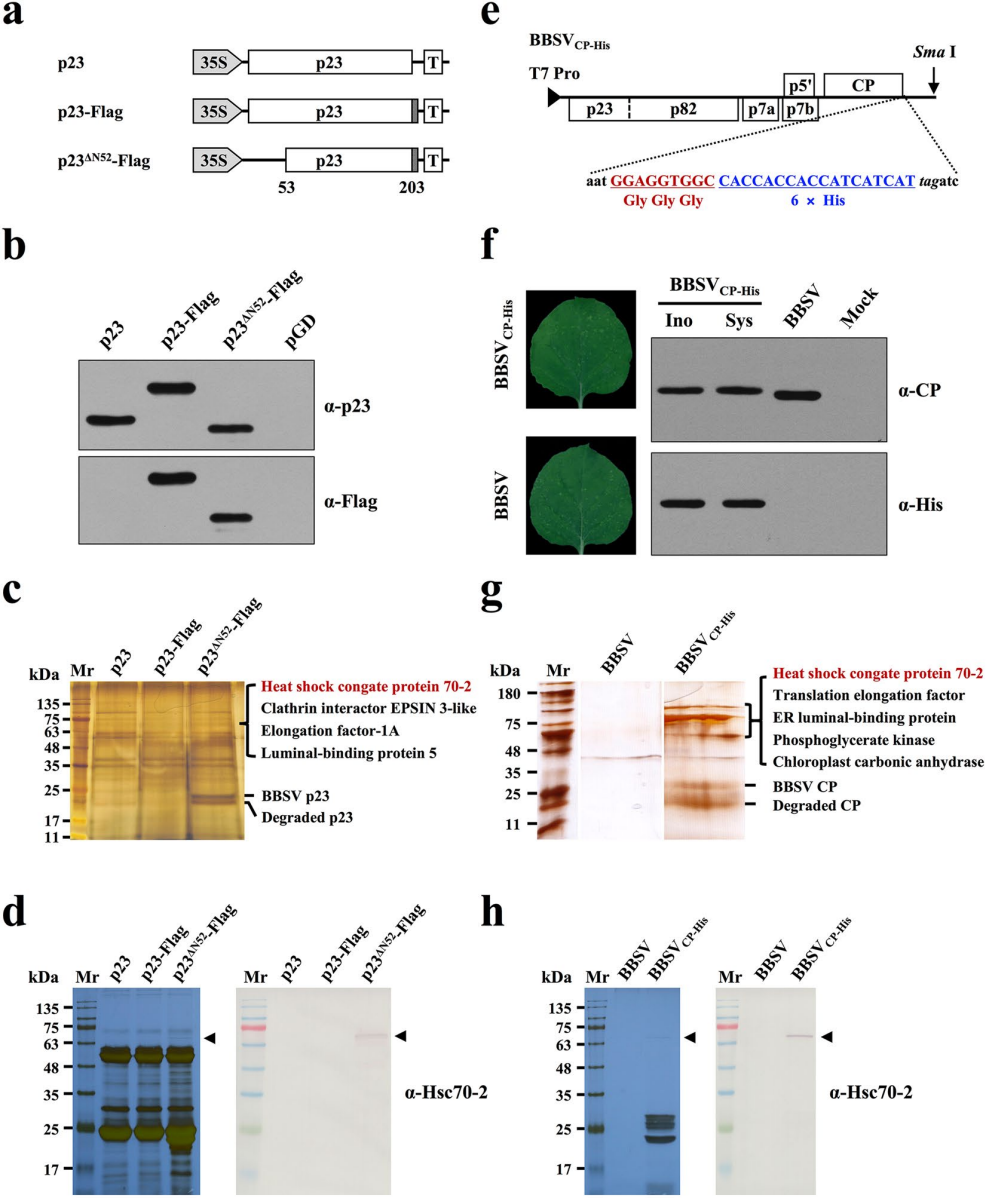
Hsc70-2 was co-purified with both BBSV p23 and CP. (**a**) Schematic representation of plasmids used for immunoprecipitation assays. Flag-tag (dotted rectangles) was engineered to the C-terminus of different p23 derivatives. (**b**) Western blot analysis of p23 and its derivatives in the infiltrated leaves of *N*. *benthamiana*. Leaves infiltrated with pGD empty vector served as the negative control. (**c**) Silver-stained SDS-PAGE gel image of p23 immunoprecipitates used for LC-MS/MS analysis. (**d**) Silver-stained SDS-PAGE and Western blot analyses of the p23 immunoprecipitates. (**e**) Schematic representation of the fusion of three glycine residues plus six His residues to the C-terminus of BBSV CP. (**f**) Analysis of the stability of His-tag during the infection of recombinant BBSV. Left panels: Symptom images of the upper uninoculated *N*. *benthamiana* leaves at 9 days after inoculation of the *in vitro* transcripts of BBSV and its derivative. Right panels: Western blot analysis of CP expression in the inoculated leaves (Ino) and upper uninoculated leaves (Sys) of *N*. *benthamiana*. Mock and wild-type BBSV-inoculated *N*. *benthamiana* plants served as negative and positive controls, respectively. (**g**) Silver-stained SDS-PAGE gel image of CP immunoprecipitates used for LC-MS/MS analysis. Note: all of the three lanes were cropped from two separate gels (see Supplementary Fig. S2). (**h**) Silver-stained SDS-PAGE and Western blot analyses of the CP immunoprecipitates.

Virus replication involves the participation of diverse host factors. To identify the host proteins that constitute the VRCs of BBSV, immunoprecipitation (IP) and liquid chromatography-tandem mass spectrometry (LC-MS/MS) analyses were performed. First, the Flag tags were fused to the C-terminus of p23 (p23-Flag); Furthermore, to increase its solubility, truncated p23 mutant that lacks the N-terminal transmembrane regions (Supplementary Fig. S1) was fused with Flag tags (p23^∆N52^-Flag) and was used for the IP experiments along with the p23-Flag (Fig. 1a), the expression of p23-Flag, p23^∆N52^-Flag, and p23 in *N*. *benthamiana* was confirmed by Western blot analysis (Fig. 1b). Next, these constructs were agroinfiltrated into BBSV-infected *N*. *benthamiana* leaves. At 3 dpi, infiltrated leaf tissues were ground in protein extraction buffer (contains detergent to solubilize membrane proteins) followed by centrifugation to remove the cell debris, the supernatant was then incubated with anti-Flag antibody-coupled agarose beads. Proteins were eluted from the Flag beads and separated by SDS-PAGE followed by silver staining. p23^∆N52^-Flag was efficiently enriched in leaf extracts, whereas the corresponding bands seem to be absent in leaf extracts expressing unmodified p23 (Fig. 1c). Additionally, p23-Flag was immunoprecipitated with low efficiency compared to that of soluble p23^∆N52^-Flag, which is possibly due to its property as a membrane protein. Differential bands between lane p23 and lane p23^∆N52^-Flag (Fig. 1c) were excised from the gel and subjected to in-gel trypsin digestions followed by LC-MS/MS analysis. BLAST results showed that several host proteins including the Hsc70-2 (Nbv5tr6412958; University of Sydney, Australia [http://sydney.edu.au/science/molecular_bioscience/benthamiana]), clathrin interactor EPSIN 3-like, elongation factor-1A, and luminal-binding protein 5 were identified (Fig. 1c). However, subsequent BiFC analyses of those four proteins showed that only Hsc70-2 interacts with p23, thus, the four host factors provided by LC-MS/MS analysis of the same band may be due to the high sensitivity of the LC-MS/MS method.

To investigate host factors that interact with BBSV CP, His tag sequences were fused to the C-terminus of CP in BBSV genome, as depicted in Fig. 1e. Mechanical inoculation of the *in vitro* transcripts of recombinant BBSV (BBSV_CP-His_) induced chlorotic spots in systemic leaves of *N*. *benthamiana*, similar to the wild-type BBSV (Fig. 1f, left panels). More importantly, BBSV_CP-His_ maintains the His-tag during infection as evidenced by the presence of His-tagged CP in the upper uninoculated leaves (Fig. 1f, right panels). Leaf extracts from *N*. *benthamiana* plants infected with wild-type BBSV or BBSV_CP-His_ were subjected to affinity purification using Ni-NTA agarose. The purified complexes were then resolved by SDS-PAGE followed by silver staining. Results showed that specific bands corresponding to the CP were presented in the BBSV_CP-His_-derived samples but not in the sample from wild-type BBSV control (Fig. 1g). Differential bands between lane BBSV and lane BBSV_CP-His_ were analyzed by LC-MS/MS as described above. Results showed that Hsc70-2 together with translation elongation factor, ER luminal-binding protein, phosphoglycerate kinase, and chloroplast carbonic anhydrase were identified within the purified CP complex (Fig. 1g). In order to show the differential bands more clearly, IP assays of p23 and CP were repeated and the differential bands were proved to be Hsc70-2 by Western blot using antibody against Hsc70-2 (Fig. 1d,h).

Taken together, these results revealed that Hsc70-2 existed in both p23^∆N52^ and CP-derived protein complexes, suggesting its potential role in BBSV infection.

### Hsc70-2 interacts with both p23 and CP *in vivo* and *in vitro*

Due to the co-purification of Hsc70-2 with p23 and CP, we used bimolecular fluorescence complementation assay (BiFC), co-immunoprecipitation (Co-IP) and GST pull-down assays to validate their interactions.

**Figure 2:**
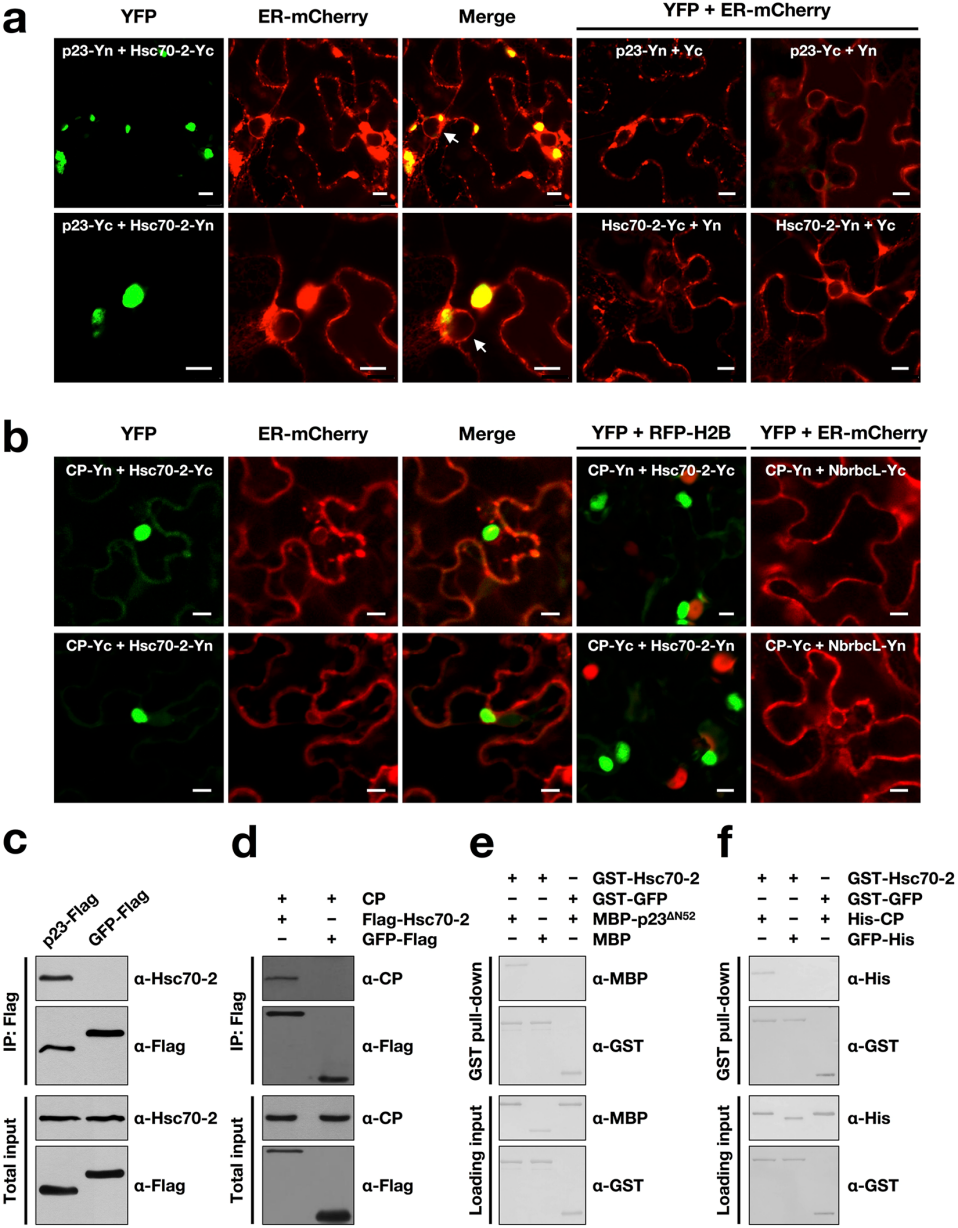
Hsc70-2 interacts with both BBSV p23 and CP. (**a**,**b**) BiFC analysis of the interactions between Hsc70-2 and p23 (**a**), and between Hsc70-2 and CP (**b**). Transient expression of mCherry-HDEL fusion was used to label the ER (ER-mCherry), and transgenic *N*. *benthamiana* expressing RFP-H2B (Histone H2B fused to RFP) was used to indicate the nucleus. NbrbcL indicates *N*. *benthamiana* RuBisCO large subunit (rbcL). White arrows point to the nuclei. Scale bars, 10 μm. (**c**,**d**) Co-IP analysis of the interactions between Hsc70-2 and p23 (**c**), and between Hsc70-2 and CP (d). (**e**,**f**) GST pull-down analysis of interactions between Hsc70-2 and p23^∆N52^ proteins (**e**), and between Hsc70-2 and CP proteins (**f**).

For BiFC, Hsc70-2 and p23 were fused with the N-terminal and C-terminal fragments of YFP respectively, and confocal microscopy was performed at 3 days post-infiltration (dpi). Results showed that either combinations of p23-Yn and Hsc70-2-Yc or p23-Yc and Hsc70-2-Yn leads to the reconstitution of YFP fluorescence (Fig. 2a), demonstrating a specific interaction between Hsc70-2 and p23 in living epidermal cells. YFP fluorescence colocalized with ER-derived aggregates, which could be induced by p23 expression alone^2^. For the negative controls, pairwise co-expression of p23-Yn and Yc, p23-Yc and Yn, Hsc70-2-Yc and Yn, Hsc70-2-Yn and Yc produced no YFP signal (Fig. 2a). The expression of various Hsc70-2 and p23 fusions in the infiltrated leaves was confirmed by Western blot analysis (Supplementary Fig. S3). The interaction between Hsc70-2 and p23 was further determined by Co-IP. *Agrobacterium* cells harboring 35S-p23-Flag were infiltrated into *N*. *benthamiana* leaves followed by leaf protein extraction at 3 dpi. The p23-Flag protein was then immunoprecipitated using the anti-Flag antibody-conjugated agarose beads followed by Western blot analysis. Consistent with BiFC results, endogenous Hsc70-2 was co-immunoprecipitated with p23-Flag (Fig. 2c). To investigate the direct interaction between Hsc70-2 and p23, recombinant GST-Hsc70-2 and MBP-p23^∆N52^ were purified from the *E*. *coli* and then subjected to the GST pull-down assay, results showed that Hsc70-2 could specifically interact with p23^∆N52^, whereas no specific band is present in either the combination of GST-Hsc70-2 and MBP or the combination of GST-GFP and MBP-p23^∆N52^ (Fig. 2e).

Likewise, the interaction between Hsc70-2 and CP was also examined by using BiFC, Co-IP and GST pull-down assays, respectively. BiFC assay showed that co-expression of both the combinations of CP-Yn and Hsc70-2-Yc or CP-Yc and Hsc70-2-Yn induced the reconstitution of functional YFP (Fig. 2b). The interaction between Hsc70-2 and CP formed large aggregates in the cytoplasm, as evidenced by the absence of colocalization with nuclear marker RFP-H2B. For the controls, pairwise co-expression of CP-Yn and NbrbcL-Yc or CP-Yc and NbrbcL-Yn produced no YFP signals in the infiltrated leaves (Fig. 2b). The expression of various Hsc70-2, CP and NbrbcL fusions in the infiltrated leaves were confirmed by Western blot analysis (Supplementary Fig. S4). Co-IP experiments indicated that CP was specifically co-precipitated by Flag-Hsc70-2 but not by GFP-Flag (Fig. 2d). The directed interaction between Hsc70-2 and CP was verified by using bait protein GST-Hsc70-2 to pull down His-CP (Fig. 2f).

Taken together, these data demonstrated that Hsc70-2 interacts with both p23 and CP *in vivo* and *in vitro*.

### Determination of the regions of Hsc70-2 that are responsible for interaction with p23 and CP

**Figure 3:**
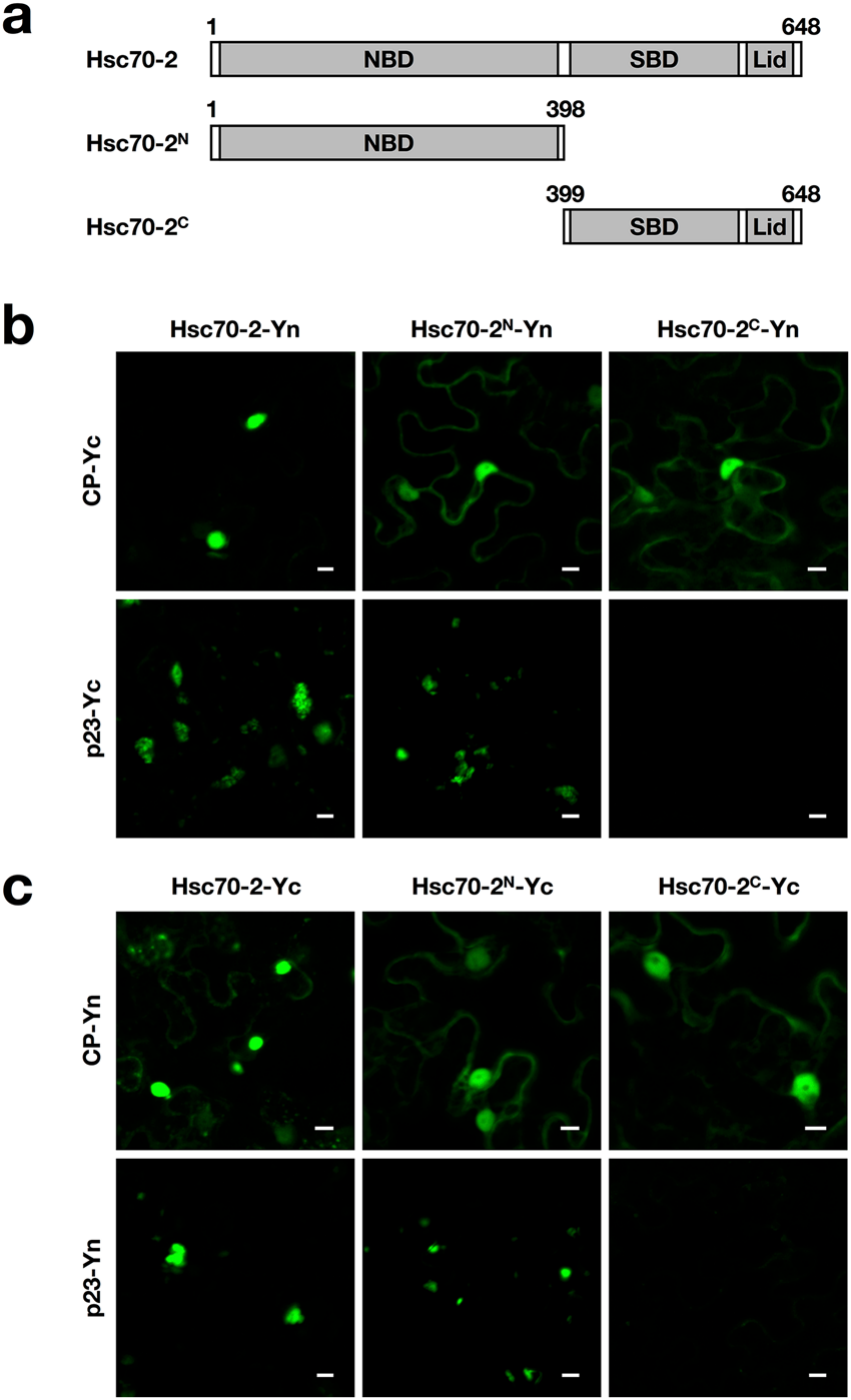
Determination of the regions within Hsc70-2 that are responsible for its interaction with CP and p23. (**a**) Schematic diagram of Hsc70-2 truncate mutants used for BiFC assays. (**b**,**c**) BiFC analysis of the interaction of N- or C-terminally truncated mutants of Hsc70-2 with CP or p23. Scale bars, 10 μm.

To determine the regions of Hsc70-2 that are responsible for interaction with CP and p23, Hsc70-2 is split into the Hsc70-2^N^ containing the N-terminal ATPase domain, and Hsc70-2^C^ consisting of the C-terminal peptide binding domain and lid domain^20^ (Fig. 3a). Interactions of Hsc70-2 and its truncation mutants with CP or p23 were analyzed by BiFC assays. The YFP fluorescence signal was observed in both combinations of Hsc70-2^N^-Yn and CP-Yc, Hsc70-2^C^-Yn and CP-Yc (Fig. 3b), and the corresponding pairwise combinations (Fig. 3c), indicating that both N-terminal and C-terminal regions of Hsc70-2 interact with the CP. In contrast, only the N-terminal region of Hsc70-2 could interact with p23 as evidenced by the YFP fluorescence emitted from the combinations of Hsc70-2^N^-Yn and p23-Yc, or Hsc70-2^N^-Yc and p23-Yn (Fig. 3b,c). The expression of the target proteins in the infiltrated leaves was validated by Western blot analysis (Supplementary Fig. S6). Collectively, these data indicated that CP and p23 had common interacting region localizing to the N-terminus of Hsc70-2, whereas the C-terminal region of Hsc70-2 interacts with the CP but not p23.

### BBSV infection slightly induces the expression of Hsc70-2

**Figure 4:**
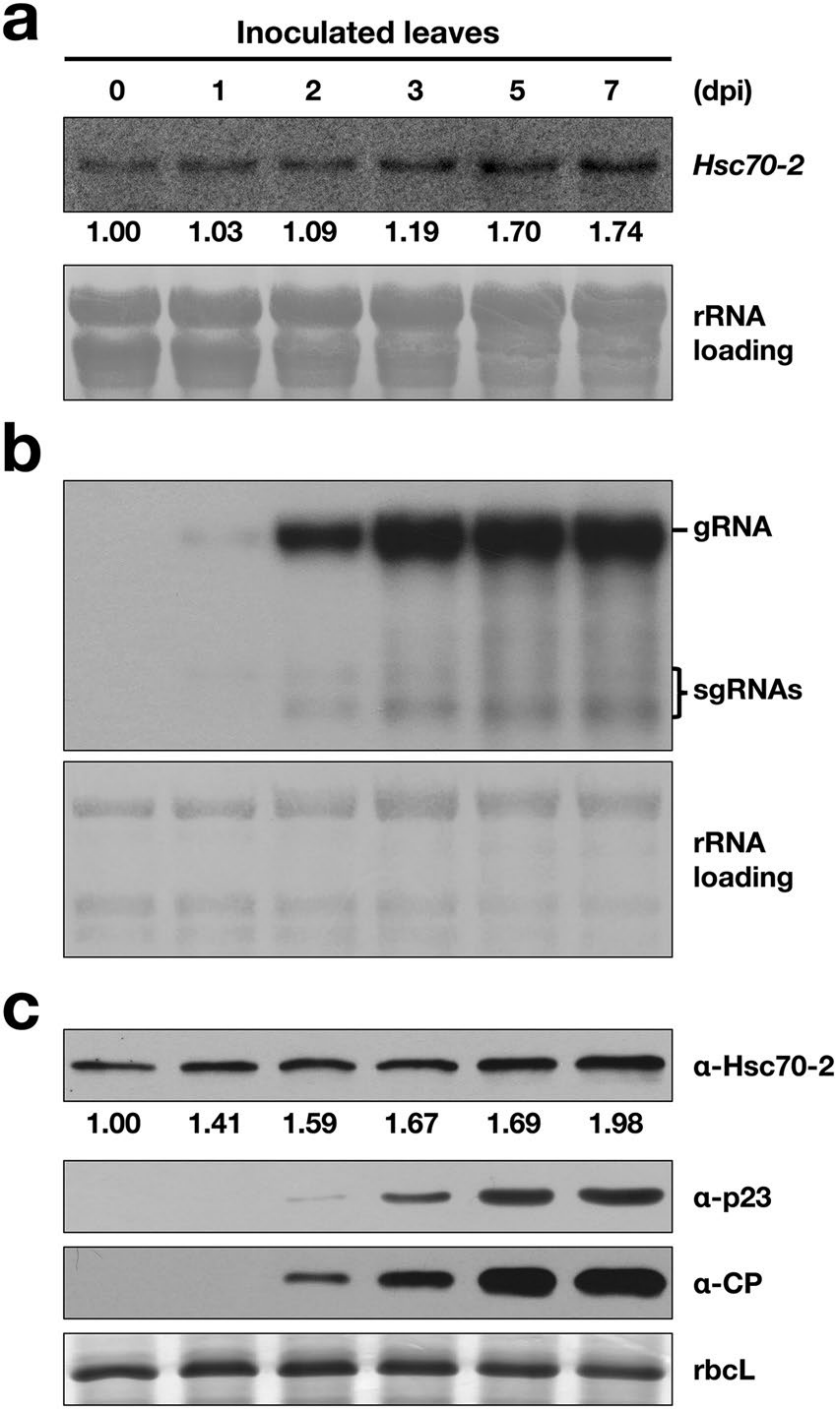
Hsc70-2 expression was elevated during BBSV infection. *N*. *benthamiana* leaves were mechanically inoculated with 100 ng BBSV virions, total RNA and protein were isolated from inoculated leaves at different time points as indicated above the panels. (**a**) Northern blot analyses of Hsc70-2 mRNA accumulations during BBSV infection. Numbers below blots represent relative abundance of each mRNA normalized to that at 0 dpi. The ribosomal (r)RNAs stained with methylene blue served as the loading control. Note: the seeming increase of rRNA loading from 0 to 7 dpi was caused by the dramatically augmented BBSV genomic RNA accumulation during ongoing viral infection, as the size of BBSV genomic RNA is just slightly larger than that of the plant 28S rRNA. (**b**) Northern blot analyses of viral RNA accumulations at different time points. (**c**) Western blot analysis of Hsc70-2, CP, and p23 in BBSV-inoculated *N*. *benthamiana* leaves at different time-points. Numbers below blots represent relative abundance of each protein normalized to that at 0 dpi. Coomassie Brilliant Blue (CBB) staining of rbcL is shown as the loading control.

To examine the effect of BBSV infection on the expression of Hsc70-2, Northern blot and Western blot were performed. Time course analysis of *N*. *benthamiana* leaves inoculated with BBSV virions showed that Hsc70-2 mRNA level was slightly up-regulated at the early stage of infection (1, 2, and 3 dpi), and was increased approximately two-fold at 7 dpi (Fig. 4a). Correspondingly, Hsc70-2 protein was increased gradually and increased about two-fold at 7 dpi compared to that at 0 dpi (Fig. 4c, top panel). RbcL loading control was measured by using Image J software (NIH) and similar values were obtained for each lane, indicating that equal amount of proteins were loaded in each lane. To exclude the effect of aging of leaves on the expression of Hsc70-2, mock- and BBSV-inoculated *N*. *benthamiana* leaves were analyzed by Western blot at different time points, the results showed the induction of Hsc70-2 expression during BBSV infection (Supplementary Fig. S7), in agreement with the results shown in Fig. 4. Meanwhile, analysis of the BBSV accumulation showed that low amounts of viral genomic RNA (gRNA) as well as the p23 and CP were produced at 1 dpi, and were dramatically increased from 2 to 7 dpi (Fig. 4b,c). Collectively, these results revealed that Hsc70-2 expression is slightly up-regulated in *N*. *benthamiana* during BBSV infection.

### Hsc70-2 is essential for BBSV replication in *N*. *benthamiana*

**Figure 5:**
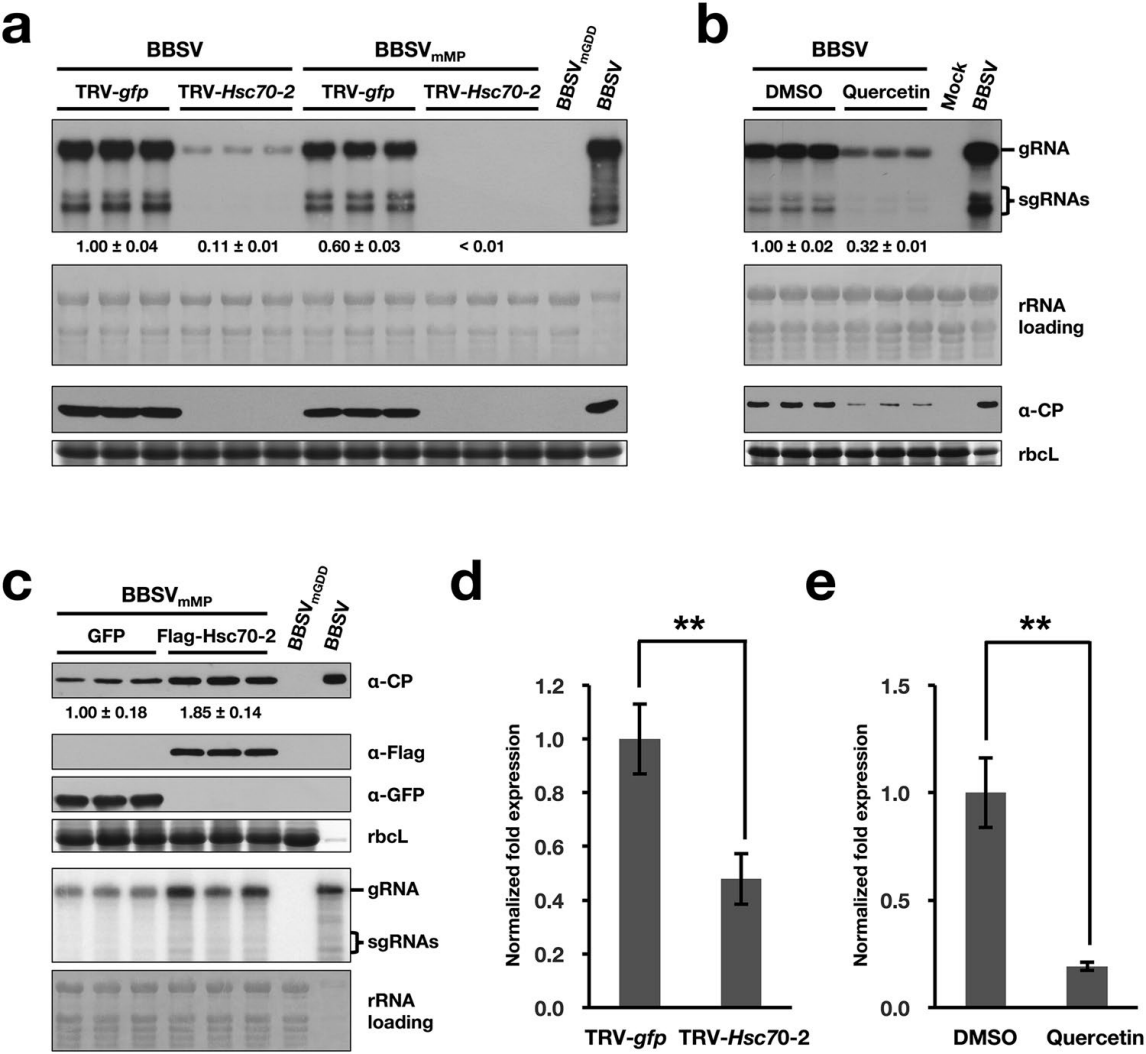
Hsc70-2 is essential for efficient accumulation of BBSV in *N*. *benthamiana* (**a**) Silencing of Hsc70-2 or Hsp70 expression in *N*. *benthamiana* impairs BBSV replication. At 7 dpi, the systemically silenced leaves from TRV-*Hsc70-2*-inoculated plants, or that from TRV-*gfp*-inoculated control plants were agroinfiltrated with wild-type BBSV or the movement-deficient BBSV mutant, BBSV_mMP_. Samples from the inoculated leaves were collected at 3 dpi for Northern blot (upper two panels) and Western blot (lower two panels) analyses. (**b**) Analysis of the BBSV accumulation in DMSO- and quercetin-treated *N*. *benthamiana* leaves. (**c**) Analysis of the BBSV_mMP_ accumulation in leaves transiently expressing GFP or Flag-Hsc70-2. Bands corresponding to gRNA and sgRNAs as well as the antibodies used for Western blot analysis in figures a, b, and c are indicated on the right. The mock-inoculated or the replication-deficient mutant, BBSV_mGDD_ served as the negative controls, *N*. *benthamiana* leaves systemically infected with BBSV served as a positive control (BBSV). Methylene blue-stained rRNAs and CBB-stained rbcL were used as RNA and protein loading controls, respectively. Numbers below blots represent relative abundance of mRNA or proteins in each treatment group normalized to that of the GFP group (n = 3 per group). (**d**) RT-qPCR was performed to confirm the silencing of Hsc70-2 in TRV-*Hsc70-2*-inoculated *N*. *benthamiana* plants (n = 6 per treatment). (**e**) RT-qPCR was performed to confirm the downregulation of Hsc70-2 expression in quercetin-treated *N*. *benthamiana* leaves (n = 3 per treatment). Error bars represent standard error (s.e.m.) of the means. Asterisks indicate statistically significant difference between the indicated groups (Student’s *t*-test, **P < 0.01).

**Figure 6:**
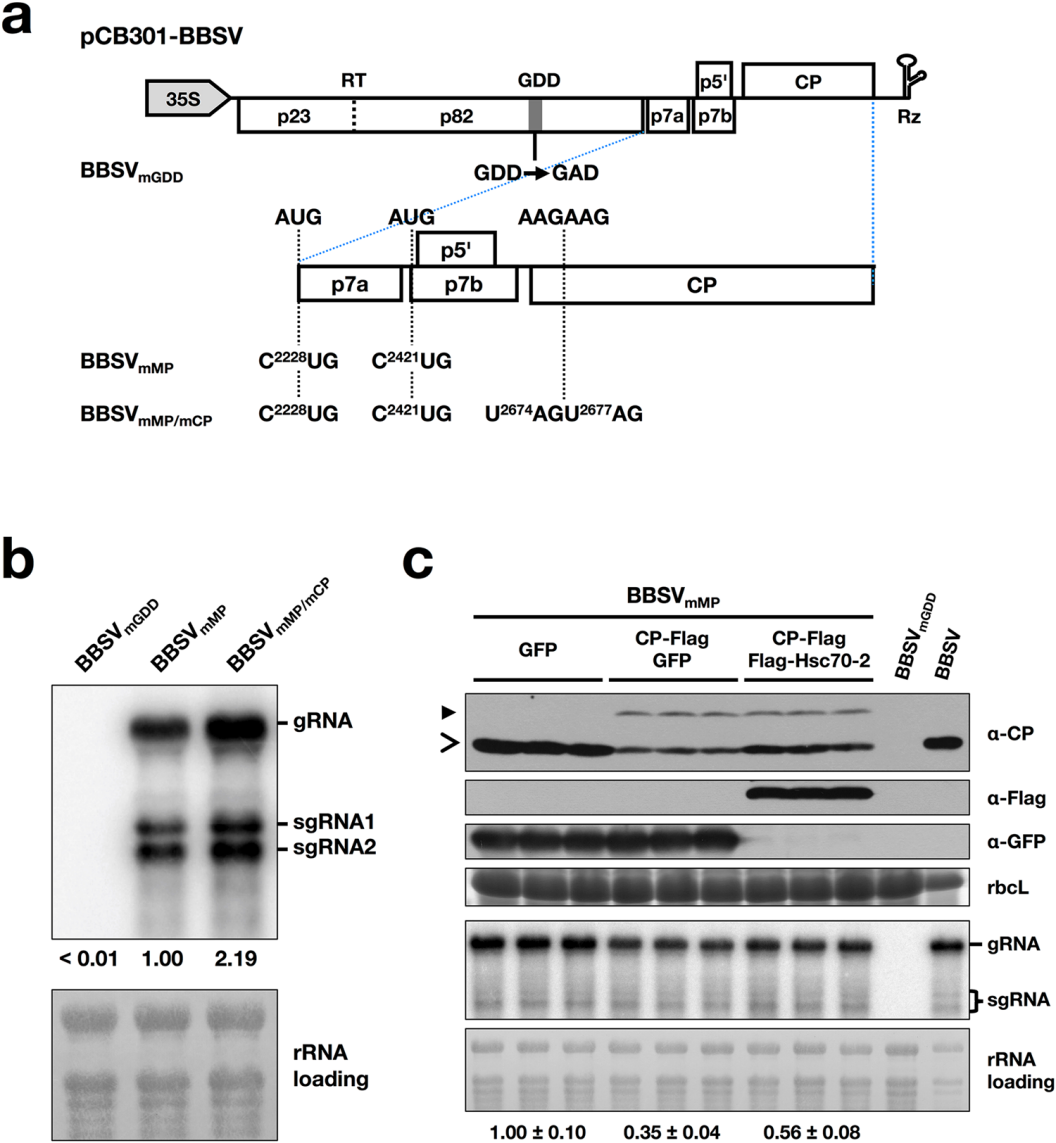
Overexpression of Hsc70-2 alleviated the inhibitory effect of CP on BBSV replication. (**a**) Schematic representation of plasmids used in *Agrobacterium tumefaciens*-mediated infection assay. Rectangles show ORFs in the BBSV genome. A stop codon read-through (RT) between p23 and p82 is illustrated by a vertical dashed line. Rz represents a *cis*-acting ribozyme. For these BBSV variants, the substitutions of these residues in the wild-type BBSV are linked by dashed lines across the expanded regions to indicate their sequence positions. The names of the mutants are indicated on the left. (**b**) Northern blot analysis of viral RNAs accumulation in *N*. *benthamiana* leaves agroinfiltrated with different BBSV-derived mutants. (**c**) Analysis of the BBSV_mMP_ accumulation in leaves transiently expressing GFP (OD_600_ = 1.0), CP-Flag (OD_600_ = 0.1) plus GFP (OD_600_ = 1.0) or CP-Flag (OD_600_ = 0.1) plus Flag-Hsc70-2 (OD_600_ = 1.0). The replication-deficient mutant, BBSV_mGDD_, served as the negative control. Bands corresponding to gRNA and sgRNAs as well as the antibodies used for Western blot analysis in figures b and c are indicated on the right. Methylene blue-stained rRNAs and CBB-stained rbcL were used as RNA and protein loading controls, respectively. Open arrow and solid arrowheads indicate bands corresponding to wild-type CP and CP-Flag, respectively. Numbers below rRNA loading panel represent relative abundance of BBSV genomic RNA in each treatment group normalized to that of the GFP group (n = 3 per group).

To investigate the role of Hsc70-2 in BBSV infection, tobacco rattle virus (TRV)-induced gene silencing (VIGS) system^42^ was used to downregulate the Hsc70-2 expression. *Hsc70-2*-silenced plants exhibited a phenotype of curling leaves, and dwarfing at 9 dpi, which become apparent at 18 dpi (Supplementary Fig. S8). Northern blot analysis indicated that the accumulations of BBSV gRNA and sgRNAs were significantly reduced in *Hsc70-2*-silenced leaves, which is in striking contrast to the high accumulation of BBSV RNAs and CP in the control TRV-*gfp*-inoculated plants (Fig. 5a). Furthermore, the replication of BBSV in *Hsc70-2*-silenced plants was evaluated by using pCB301-BBSV_mMP_, a movement-deficient mutant BBSV (see the diagram in Fig. 6a). As shown in Fig. 5a, the accumulations of viral RNAs and CP were much lower in *Hsc70-2*-silenced plants than that in unsilenced ones. Overexposure image of the same blot showed that both BBSV and BBSV_mMP_ can infect *Hsc70-2*-silenced leaves as evidenced by the specific band signals corresponding to viral RNAs (Supplementary Fig. S9). Moreover, when quercetin, a flavonoid that is used to inhibit *Hsp70* gene expression in *N*. *benthamiana*^43^, was employed to investigate the role of Hsc70-2 in BBSV infection, results showed that both BBSV RNAs and CP accumulations were substantially decreased in the quercetin-treated leaves in comparison to the DMSO control (Fig. 5b), consistent with the dramatic reduction of BBSV accumulation in TRV-*Hsc70-2*-inoculated plants. Due to the high nucleotide sequence similarity among different Hsp70 isoforms, knock-down of other *Hsp70* isoforms besides *Hsc70-2* in the TRV-mediated silencing assay or after quercetin treatment could not be absolutely excluded. Despite this, the results shown above could, at least to a large extent, reflect the essential role of Hsc70-2 in BBSV replication. The efficient downregulation of *Hsp70* or *Hsc70-2* by the TRV-based VIGS system and quercetin treatment was confirmed by RT-qPCR (Fig. 5d,e). Collectively, these results demonstrated that *Hsp70* or *Hsc70-2* is essential for BBSV replication.

To investigate whether overexpression of Hsc70-2 could promote BBSV replication, *N*. *benthamiana* leaves were co-infiltrated with *Agrobacterium* containing pCB301-BBSV_mMP_ and pSuper1300-Flag-Hsc70-2 (or pSuper1300-GFP) at an OD_600_ of 0.1 and 1.0, respectively. Samples were collected at 3 dpi and subjected to Western blot and Northern blot analysis. The results showed that the accumulations of both viral CP protein and viral RNAs were enhanced in leaves transiently expressing Flag-Hsc70-2 compared to that expressing the GFP control (Fig. 5c), indicating that overexpression of Hsc70-2 facilitates BBSV replication.

### The inhibitory effect of CP on BBSV replication is alleviated by overexpression of Hsc70-2

A growing body of evidence shows that viral CP is a multi-functional protein with roles outside of virus packaging^15,44^. To investigate what role of BBSV CP in virus replication, three different viral clones were constructed as depicted in Fig. 6a: pCB301-BBSV_mGDD_, which destroys the RdRp activity; pCB301-BBSV_mMP_, which retains BBSV replication but cannot undergo cell-to-cell movement; and pCB301-BBSV_mMP/mCP_, which loses both cell-to-cell movement and CP expression (Fig. 6a). *Agrobacterium* cells carrying one of these constructs were infiltrated into *N*. *benthamiana* leaves. Northern blot was performed at 2 dpi using BBSV 3′-UTR-specific probes. Results showed that BBSV_mMP/mCP_ accumulated with a higher level than that of BBSV_mMP_ (Fig. 6b), indicating the negative effect of CP on BBSV replication.

To investigate whether CP regulates BBSV replication by directly interacting with the replication proteins, BiFC assay was performed. Results showed that neither p23 nor p82 interacts with CP in healthy or BBSV-infected leaves (Supplementary Fig. S10). Due to the interaction of Hsc70-2 with both p23 and CP, we presume that CP-mediated inhibition of BBSV replication might involve the participation of Hsc70-2. To examine this hypothesis, *N*. *benthamiana* leaves were agroinfiltrated with pCB301-BBSV_mMP_ and pCP-Flag along with pSuper1300-Flag-Hsc70-2 or pSuper1300-GFP. Northern blot and Western blot assays were performed at 3 dpi. Results showed that the accumulations of BBSV_mMP_ gRNA and CP were decreased in leaves transiently expressing CP-Flag and GFP in comparison to that expressing the GFP alone (Fig. 6c), consistent with the results described in Fig. 6b. Furthermore, the accumulations of BBSV_mMP_ RNAs and CP in leaves coexpressing CP-Flag and Hsc70-2 were higher than those coexpressing CP-Flag and GFP, while still lower than those expressing GFP alone (Fig. 6c). These results indicate that overexpression of Hsc70-2 can alleviate the inhibitory effect of CP on BBSV replication. We noted that the difference in the accumulations of BBSV_mMP_ sgRNAs among indicated groups is not obvious, a similar phenomenon was also observed during analysis of BaMV RNA accumulations^40^. The discrepancy between genomic RNA and sgRNAs accumulation changes may be due to the different mechanisms underlying their production.

### CP impairs the interaction between p23 and Hsc70-2 in a dose-dependent manner

**Figure 7:**
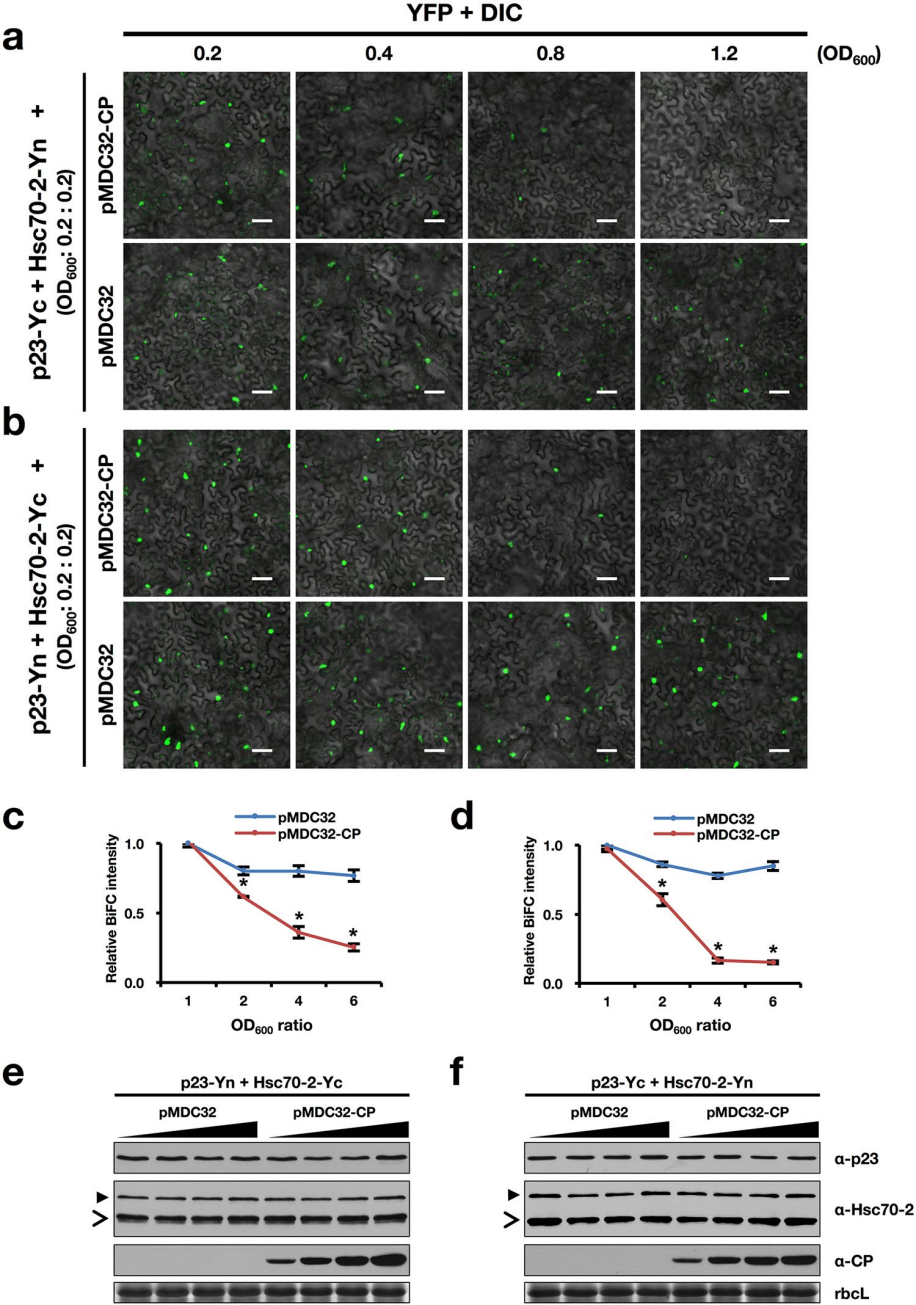
BBSV CP interferes with the interactions between Hsc70-2 and p23 in a dose-dependent manner. (**a**,**b**) Competitive BiFC analysis of the effect of CP on the interactions between Hsc70-2 and p23. Different combinations of constructs used for agroinfiltration are indicated on the left. Scale bars, 50 μm. Concentrations of *Agrobacterium* cells carrying pMDC32 or pMDC32-CP were indicated above the panels. (**c**,**d**) Graphical representation of the relative BiFC intensity as shown in (**a**) and (**b**). YFP fluorescence for each panel was measured using Image J software (NIH), and relative BiFC intensity was diagramed by normalizing the fluorescence intensity of each combination to that of the pMDC32-CP or pMDC32 with the lowest concentration (OD_600_ = 0.2). The y-axis shows the relative BiFC intensity, the x-axis indicates the ratio of different concentrations of *Agrobacterium* cells relative to that of OD_600_ = 0.2. Error bars represent standard deviation from the mean (n = 3). Asterisk indicates statistically significant difference between pMDC32 and pMDC32-CP groups under the same OD_600_ ratio (Student’s *t*-test, *P < 0.05). (**e**,**f**) Western blot analysis of total protein extracts from agroinfiltrated leaves corresponding to (**a**) and (**b**). The open arrowheads point to the bands of endogenous Hsc70-2, whereas solid arrowheads indicate the split YFP-fused Hsc70-2. CBB staining of rbcL is shown as the loading control.

Considering the repression of BBSV replication by the CP and the overlapping interacting region of Hsc70-2 responsible for interactions with p23 and CP, we assumed that CP might impact the interaction between p23 and Hsc70-2. Hence, equal amounts (OD_600_ = 0.2) of *Agrobacterium* cells carrying pSPYCE-p23 and pSPYNE-Hsc70-2, together with increasing concentrations (OD_600_ = 0.2, 0.4, 0.8, 1.2) of *Agrobacterium* cells harboring pMDC32-CP, were co-infiltrated into *N*. *benthamiana* leaves. Confocal microscopic analysis was carried out at 3 dpi. YFP signal was found to decrease with increasing concentrations of pMDC32-CP-containing *Agrobacterium*, while control *Agrobacterium* harboring the empty vector (pMDC32) had a minimal effect on the interaction between Hsc70-2 and p23 (Fig. 7a,c). Similar results were obtained for the pairwise combination of p23-Yn and Hsc70-2-Yc (Fig. 7b,d). Similarly, competitive Co-IP assay was carried out to examine the effect of p23 on CP-Hsc70-2 interaction, results showed that overexpression of p23 has little, if any, effect on the interaction between CP and Hsc70-2 (Supplementary Fig. S11). Furthermore, expression of p7a, the movement protein of BBSV^45^, also showed no marked influence on the interaction between p23 and Hsc70-2 (Supplementary Fig. S12), substantiating the specificity of impairing effect of CP on the interaction of Hsc70-2 with p23. Additional Western blot analysis confirmed the invariable expression of p23 and Hsc70-2 under the treatment of different concentrations of *Agrobacterium* cells carrying pMDC32-CP or the control empty vector pMDC32 (Fig. 7e,f). Collectively, these results indicated that CP interferes with the interaction between p23 and Hsc70-2 in a dose-dependent manner.

## Discussion

Hsp70 chaperones are one of the major constituents of the cellular protein quality control system and are often employed by viruses at several stages of their life cycle. Various studies have shown that Hsp70 family proteins associate with VRCs and enhances viral RNA replication^41^. For example, Hsp72 has been shown to interact with hepatitis C virus (HCV) replication proteins NS5A, NS3 and NS5B, and function as a positive regulator of HCV replication^46^. For tombusvirus, Hsp70 promotes the subcellular transport of replication proteins to intracellular membranes and is required for the *in vitro* activity or assembly of the VRCs^36,37^. Many distantly related viruses recruit Hsp70 to the membranous replication factories, such as RCNMV^47^, turnip mosaic virus (TuMV)^48^, and Chinese wheat mosaic virus (CWMV)^49^. In this study, Hsc70-2 was copurified with BBSV auxiliary replication protein p23 (Fig. 1c,d), and their interaction occurs in the ER-derived aggregates (Fig. 2a), where BBSV replication occurs^2^. Additionally, the expression level of Hsc70-2 is changed during BBSV infection (Fig. 4), and disruption of Hsc70-2 expression severely impedes BBSV replication (Fig. 5). These results consistently suggest that Hsc70-2 is recruited by the auxiliary replication protein p23 to the VRCs and plays an essential role in BBSV replication.

The function of Hsp70s in the assembly of virus particles had been widely characterized in many viruses^27,32,33,50^. Recently, Alam *et al*. reported that Hsc70-2 binds to purified CNV virions and plays a role in disassembly of virus particles^39^. Hsc70 was also reported to participate in the intercellular transport of pepino mosaic virus (PepMV)^25^. In this study, BiFC analysis showed that Hsc70-2 binds to BBSV CP through its N-terminal and C-terminal regions (Fig. 3b,c). Interestingly, YFP fluorescence indicative of CP-Hsc70-2^N^ or CP-Hsc70-2^C^ interactions presents a nucleocytoplasmic distribution, which is distinct from the cytoplasmic pattern displayed by CP-Hsc70-2 interactions (Fig. 2b). This is likely due to the destruction of the functional integrity of Hsc70-2.

In this study, CP was found to negatively regulate the replication of BBSV (Fig. 6b,c), the similar phenomenon was also observed in TNV-D which also belongs to the genus *Betanecrovirus*^16^. Further BiFC analysis showed that neither BBSV auxiliary replication protein p23 nor the polymerase p82 binds to the CP (Supplementary Fig. S10), excluding the possibility that CP regulates BBSV replication by directly interacting with viral replication proteins. It has been reported that brome mosaic virus (BMV) CP negatively regulates viral replication by binding to the RNA elements within the 5′-UTR, leading to the suppression of viral replication proteins translation^17,18^. Our previous work indicated that BBSV CP binds to the viral RNA via its N-terminal region^5^. We therefore examined the effect of the RNA binding activity of CP on BBSV replication. Results showed that the BBSV_mMP/CP_^ΔN21^ mutant showed a similar accumulation of viral RNAs to that of BBSV_mMP_, suggesting that the inhibitory effect of CP on BBSV replication was not significantly affected by inactivating the RNA binding activity within the CP (Supplementary Fig. S13), in contrast to the elevated accumulation of BBSV RNAs when the whole CP expression was destroyed as shown in Fig. 6b. However, CP^ΔN21^ maintains the ability to interact with Hsc70-2 (Supplementary Fig. S13). These results further support the association of CP-mediated inhibition of BBSV replication with its impact on the p23-Hsc70-2 interaction, which is somewhat distinct from the functional role of BMV CP in regulating viral RNA accumulation^17,18^.

Our findings that the N-terminus of Hsc70-2 could interact with both p23 and CP (Fig. 3) raises the possibility that CP competed with p23 for available Hsc70-2. Since CP becomes the most abundant protein during virus infection, it should have an advantage in contacting Hsc70 molecules, resulting in less Hsc70 available for p23 binding, leading to the failure of forming functional VRCs. Our results support this hypothesis by showing that overexpression of Hsc70-2 alleviated the inhibitory effect of CP on BBSV replication (Fig. 6c), and increasing CP expression levels interfered with the interaction of Hsc70-2 with p23 (Fig. 7). Although Hsc70-2 was induced during BBSV infection, the changes of Hsc70-2 expression were relatively mild in comparison to the drastic increase in viral CP accumulation (Fig. 4). Thus, it is reasonable to propose that large amounts of CP protein produced during ongoing viral infections would extend well beyond the ability that the moderately augmented amounts of Hsc70-2 could rescue. Collectively, these results suggest that Hsc70-2 may serve as a limiting factor hijacked by different BBSV components to fine-tune the BBSV infection.

In summary, this study demonstrates that Hsc70-2 interacts with BBSV CP and p23, and is essential for BBSV replication. CP negatively regulates the replication of BBSV, which can be mitigated by overexpression of Hsc70-2. High concentration of CP disturbs the interaction between p23 and Hsc70-2, which may impede the formation of functional VRCs. These data broaden our understanding of the diverse roles of Hsp70 family proteins in virus infection.

## Methods

### Plant material and growth conditions

*N*. *benthamiana* plants were maintained in the growth chamber at 24 °C or 18 °C on a 16/8 h light/dark cycle.

### Plasmid construction

To construct the BBSV infectious clone pCB301-BBSV for *Agrobacterium tumefaciens*-mediated infection assays, the full-length cDNA of BBSV was cloned into the binary vector pCB301-2 × 35S-MCS-HDVRz-NOS using the Seamless Assembly Cloning Kit from Clone Smarter. BBSV mutants used in this study were generated from pCB301-BBSV. Replication-deficient BBSV (BBSV_mGDD_) was constructed by mutating the GDD motif of p82 to GAD, and movement-deficient BBSV (BBSV_mMP_) was constructed by mutating AUG start codons of p7a and p7b to CUG, respectively. MP and CP double mutant BBSV_mMP/mCP_ was constructed by further mutating ^28^AAGAAG^33^ to ^28^TAGTAG^33^ in the CP ORF of BBSV_mMP_. To engineer the His-tag to the C-terminus of CP in BBSV genome, three linked glycine residues and 6 × His-tag were fused to the C-terminus of CP to yield the intermediate plasmid pCPmx-7_His_^1^. For pUBF52-CP_His_, the DNA fragment encoding the His-tagged CP (CP-His) were excised from the plasmid pCPmx-7_His_, and used to replace the counterpart in the plasmid pUBF52, the infectious cDNA clone of BBSV^4^ (Fig. 1e).

To construct pMDC32-p23-Flag and pMDC32-p23^∆N52^-Flag for immunoprecipitation assay, p23 and p23^∆N52^ (lacking the p23 transmembrane domain) were amplified from pUBF52, and cloned into the binary vector pMDC32-Flag, a modified version of pMDC32^51^. The DNA fragment encoding three linked glycine residues and three tandem repeats of Flag epitopes (3 × Flag) were fused to the N-terminus of Hsc70-2 to yield plasmid pMD19T-3 × Flag-Hsc70-2. To generate pMDC32-3 × Flag-Hsc70-2, the 3 × Flag-Hsc70-2 fragment was amplified, introduced into pDONR/Zeo entry plasmid (Invitrogen) by Gateway LR recombination, and transferred into pMDC32 destination vector^51^ by BP recombination. The *gfp* gene was cloned into the plasmid pMDC32-Flag using standard molecular cloning methods^52^ to generate pMDC32-GFP-Flag.

For transient expression assay, the 3 × Flag-Hsc70-2 gene fragment was amplified from pMD19T-3 × Flag-Hsc70-2, and cloned into pSuper1300-Myc^53^ using the Seamless Assembly Cloning Kit from Clone Smarter. DNA fragments encoding CP or CP-Flag were amplified from pUBF52 using Phusion HF DNA polymerase (NEB), followed by cloning of the fragments into pMDC32^51^.

For protein expression, the DNA fragment encoding the *N*. *benthamiana* Hsc70-2 was amplified, digested, and cloned into the pGEX-KG vector. p23^∆N52^ gene was cloned into pMAL-c2x (NEB) using the Seamless Assembly Cloning Kit from Clone Smarter. The *gfp* gene was cloned into pET-30a(+) (Novagen) and pGEX-KG vectors using standard molecular cloning methods.

For BiFC assay, the *Hsc70-2* gene and its truncated mutants (Hsc70-2^N^ and Hsc70-2^C^) were amplified and cloned into binary vectors pSPYNE-35S and pSPYCE-35S^54^. For virus-induced gene silencing assay, a 360 bp fragment amplified from the gene of *Hsc70-2* was cloned into the plasmid pTRV2^42^; a *gfp* gene fragment was inserted into pTRV2 to serve as the negative control.

All primer pairs used for plasmid construction are listed in Supplementary Table S1, and all the constructs were sequenced to confirm their accuracy.

Several plasmids (pSPYCE-p23, pSPYNE-p23, pSPYCE-NbrbcL, pSPYNE-NbrbcL, pGD-p23, pSPYCE-CP, pSPYNE-CP, pET-30a(+)-CP, and pSuper1300-GFP) used in this study were described in previous studies^2,6,53,55^.

### Preparation of Hsc70-2-specific antibody

Hsc70-2-His protein was expressed and purified from *E*. *coli* strains Rosetta and then sent to Beijing Protein Innovation Center for the preparation of rabbit polyclonal antibody against Hsc70-2.

### Mass spectrometry analysis

Immunoprecipitated samples were separated by SDS-PAGE followed by silver staining. Target bands were excised from the gel and digested with trypsin at 37 °C. The digested peptides were separated by nanoscale C18 reverse phase liquid chromatography (Waters Associates) and electro-sprayed into a Q-Exactive mass spectrometer (Thermo Fisher Scientific) at the Mass Spectrometry Facility of China Agricultural University. Protein identification was performed by searching against the protein database of National Center for Biotechnology Information (NCBI).

### Co-immunoprecipitation (Co-IP) assay

Co-IP was performed according to previously described methods^56^ with minor modifications. Briefly, 4 g *N*. *benthamiana* leaves was ground in liquid nitrogen and thawed on ice in two volumes (w/v) of extraction buffer containing 50 mM Tris-HCl (pH 7.5), 150 mM NaCl, 1 mM EDTA, 10% (v/v) glycerol, 2% (w/v) polyvinylpyrrolidone, 10 mM dithiothreitol (DTT), 1 × protease inhibitor cocktail, and 0.1% (v/v) Tween 20. After centrifugation, the supernatant was incubated with anti-Flag M2 affinity Gel (Sigma-Aldrich) at 4 °C for 5 hr on a rotator. The resin was washed with IP buffer [50 mM Tris-HCl (pH 7.5), 150 mM NaCl, 1 mM EDTA, 10% (v/v) glycerol, and 0.1% (v/v) Tween 20], and eluted by incubation with IP buffer containing 0.17 mg/mL Flag peptides (Sigma-Aldrich). The input and eluate were then analyzed by Western blot. Because p23 is a membrane protein, the extraction buffer for Co-IP analysis of p23-Hsc70-2 interaction was modified as follows: 50 mM Tris-HCl (pH 7.5), 150 mM NaCl, 1 mM EDTA, 10% (v/v) glycerol, 2% (w/v) polyvinylpyrrolidone, 10 mM dithiothreitol (DTT), 1 × protease inhibitor cocktail, 1% NP-40 and 0.1% (w/v) SDS.

### Expression and purification of recombinant proteins

Protein purification was performed as described previously with minor modification^6^. The plasmid was introduced into *E*. *coli* strains BL21 (DE3) or Rosetta. The bacteria were incubated at 37 °C to an OD_600_ of 0.8 followed by induction with 0.2 mM IPTG (Sigma) for 18 hr at 18 °C. Cells were collected by centrifugation and resuspended in column buffer [20 mM Tris-HCl (pH 7.3), 500 mM NaCl, 10% (v/v) glycerol, 0.1% (v/v) Triton X-100, 1 mM PMSF]. The bacteria were disrupted by sonication and then centrifuged at 20,000 *g* for 30 min. Recombinant protein in the supernatant was purified using Glutathione Sepharose 4 Fast Flow (GE Healthcare), Ni-NTA agarose (QIAGEN) or amylose resin (NEB) according to the manufacturer’s instructions.

### GST pull-down assay

GST pull-down assay was conducted as described previously with minor modifications^57^. About 10 μg of purified prey protein was pre-absorbed for 1.5 hr at room temperature in 1 mL binding buffer containing 15 μL of Glutathione Sepharose 4 Fast Flow (GE Healthcare), 50 mM Tris-HCl (pH 7.5), 250 mM NaCl, 0.6% (v/v) Triton X-100, 1 × protease inhibitor cocktail, 0.2% (v/v) glycerol, and 5 mM DTT. After centrifugation, the supernatant was incubated with 10 μg of bait protein and another 15 μL Glutathione Sepharose 4 Fast Flow (GE Healthcare) for 3 hr at room temperature. The column was then washed with buffer containing 50 mM Tris-HCl (pH 7.5), 250 mM NaCl, 0.6% (v/v) Triton X-100, and protease inhibitor cocktail, bound proteins were eluted by boiling in 60 μL SDS-containing buffer and subjected to Western blot analysis.

### Agroinfiltration

Agro-infiltration was described in the previous report^58^. Briefly, the plasmid was introduced into cells of *Agrobacterium tumefaciens* strains EHA105 or GV3101 by a freeze-thaw method as previously described^59^. Selected colonies were cultured in LB medium containing 100 μg/mL kanamycin and 25 μg/mL rifampicin at 28 °C for 14-16 hr. *Agrobacterium* cells were harvested and resuspended in infiltration media [10 mM MgCl_2_, 150 μM acetosyringone, and 10 mM MES (pH 5.6)]. A needle-free syringe was used for agroinfiltration of *N*. *benthamiana* leaves.

### Confocal laser scanning microscopy

Agroinfiltrated *N*. *benthamiana* leaves were observed using a Zeiss LSM710 confocal microscope equipped with Zeiss Zen 2012 software. GFP, YFP and RFP fluorescence was visualized under 488 nm, 514 nm, and 543 nm respectively with an argon laser. Images were captured digitally with a Zeiss Axiocam camera and processed with Imaris 7.4.2 software (Bitplane). Sequential scanning mode was used to avoid crosstalk between neighboring GFP and YFP emission spectra.

### Inhibition of Hsc70-2 expression

TRV-based VIGS system and the Hsp70-specific chemical inhibitor quercetin (Sigma-Aldrich) were used to downregulate Hsp70 or Hsc70-2 expression in *N*. *benthamiana*.

For VIGS, *A*. *tumefaciens* cells harboring pTRV2-gfp or pTRV2-Hsc70-2 was mixed with *A*. *tumefaciens* cells containing pTRV1^42^, and then infiltrated into *N*. *benthamiana* leaves at the four-leaf stage. *N*. *benthamiana* plants were maintained in the growth chamber at 25 °C for seven days until silencing of Hsp70 or Hsc70-2 occurred. The systemically silenced leaves were then agroinfiltrated with pCB301-BBSV or its derivatives at an OD_600_ of 0.1, followed by maintenance in another growth chamber at 18 °C^60^.

Quercetin (Sigma-Aldrich) was used to inhibit *N*. *benthamiana* Hsp70 or Hsc70-2 expression as reported previously^61^. Briefly, 200 mM stock solution was prepared by dissolving the quercetin in dimethyl sulfoxide (DMSO) and was further diluted to 1 mM in 10 mM Na_2_CO_3_-NaHCO_3_ buffer (pH 9.6) prior to infiltration of leaves. Equal concentration of DMSO was infiltrated into leaves to serve as the negative control. After 1 hr, 300 ng BBSV virions were inoculated onto the infiltrated leaves. Leaf samples were collected at 3 dpi and subjected to molecular analysis.

### Northern blot

Northern blot analyses were performed as described previously with minor modifications^62^. In brief, total RNA was denatured at 60 °C for 10 min followed by electrophoresis on the 1.2% (w/v) agarose gel under denaturing conditions. RNA was vacuum-blotted onto a Hybond-N^+^ membrane (GE Healthcare) and hybridized with α-^32^P-labeled DNA probes corresponding to nt 2647-3644 of BBSV gRNA. The membranes were washed and exposed to a phosphorimager screen (GE Healthcare) for 24 hr, and the hybridization signals were visualized with a Typhoon 9410 scanner (GE Healthcare) and analyzed with ImageQuant software (version 5.2).

### Reverse transcription quantitative real-time PCR (RT-qPCR)

RT-qPCR was performed according to previously described methods^63^. First-strand cDNA was synthesized using the M-MLV Reverse Transcriptase (Promega). *Elongation factor 1α* (*EF-1α*) gene was used as an internal control. All primer pairs used for RT-qPCR analyses are listed in Supplementary Table S1.

### Data availability statement

No datasets were generated or analysed during the current study.

## Electronic supplementary material

Supplementary Information
