# Supplementary material for: Hsc70-2 is required for Beet black scorch virus infection through interaction with replication and capsid proteins
Source: Sci Rep. 2018 Mar 14;8:4526. doi: 10.1038/s41598-018-22778-9 (PMC5852052; doi:10.1038/s41598-018-22778-9)
Supplement: Supplementary file 1 — Supplementary Information [file 41598_2018_22778_MOESM1_ESM.pdf]

Hsc70-2 is required for *Beet black scorch virus* infection through interaction with replication and capsid proteins

Xiaoling Wang[1+], Xiuling Cao[1+], Min Liu[1], Ruiqi Zhang[1], Xin Zhang[1], Zongyu Gao[1], Xiaofei Zhao[1], Kai Xu[2], Dawei Li[1] & Yongliang Zhang[1]*

Supplementary Information

Supplementary Figures

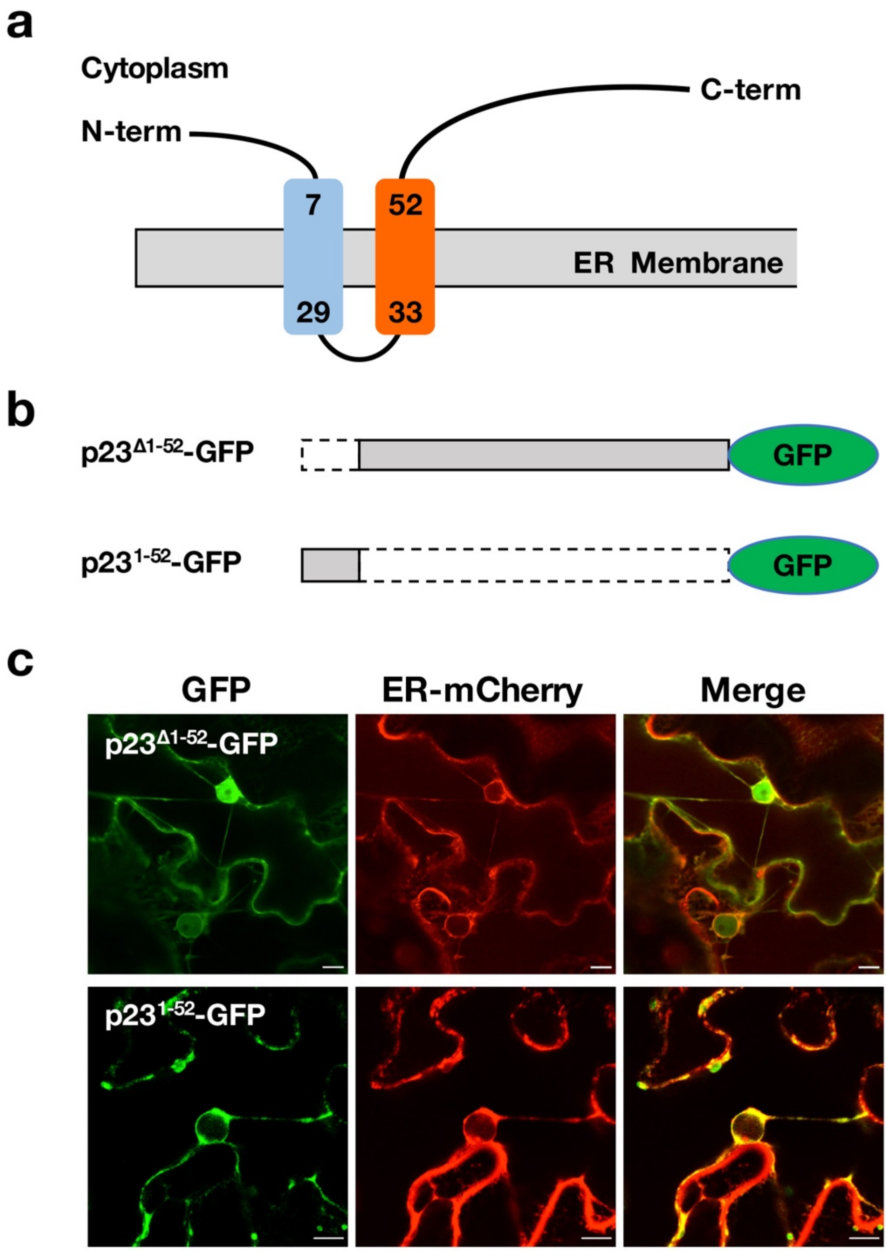

Supplementary Figure S1. The N-terminal 52 amino acids contain transmembrane region of p23. **(a)** Schematic illustration of p23 orientation in ER membrane. The N-terminal two transmembrane helices are shown as bars traversing the bilayer membrane. **(b)** Diagram of the N-terminal and C-terminal truncation mutants of p23 that were fused with GFP. **(c)** Subcellular localization of $p23^{\Delta 1\text{-}52}$-GFP and $p23^{1\text{-}52}$-GFP in agroinfiltrated leaves of *N. benthamiana* at 3 dpi. Scale bars, 10 µm.

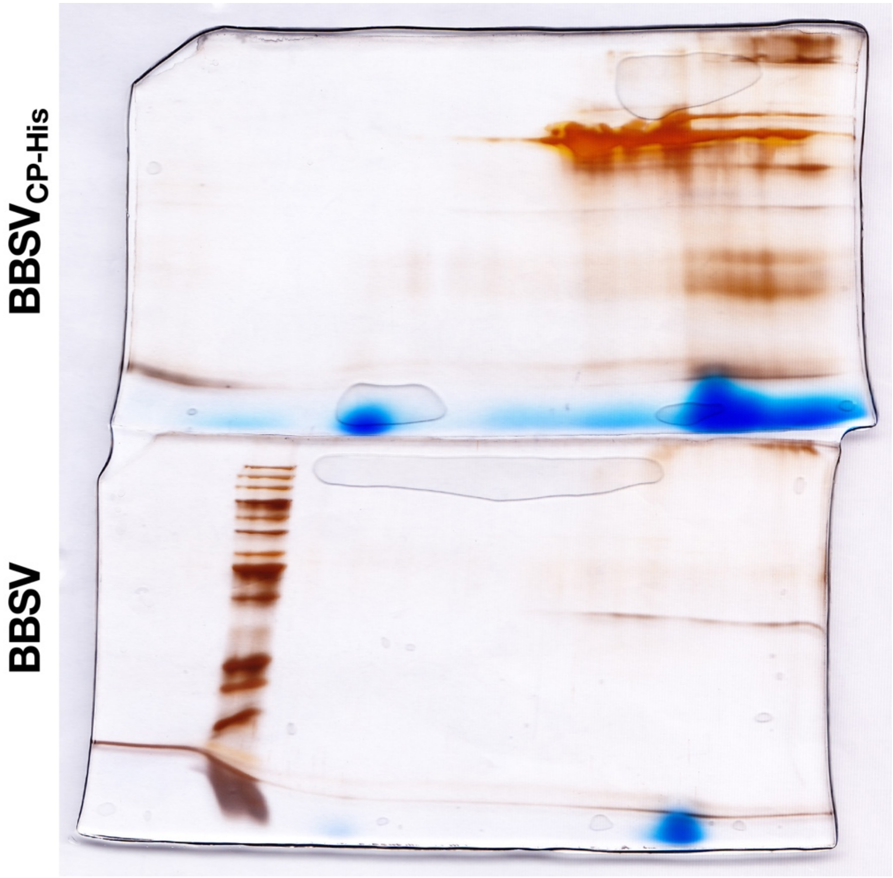

Supplementary Figure S2. Original images of silver-stained polyacrylamide gels shown in Figure 1g.

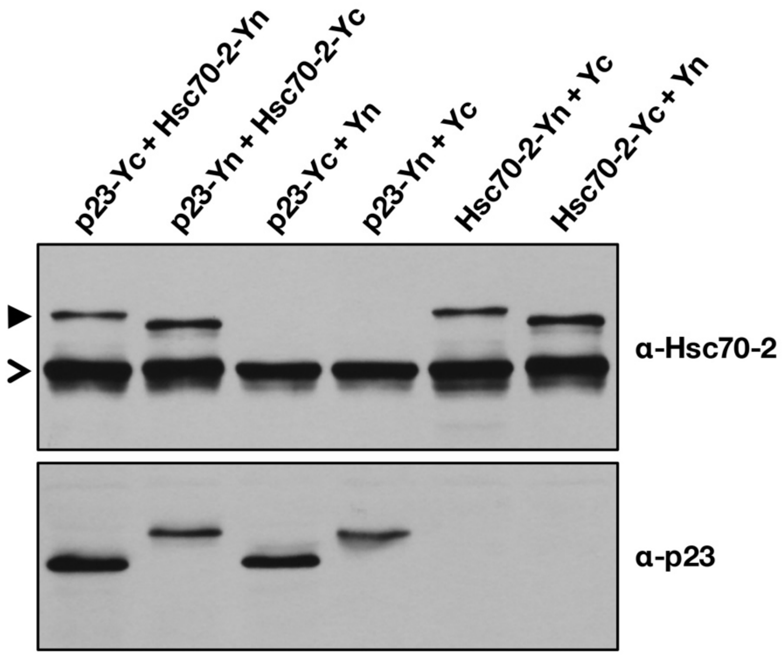

Supplementary Figure S3. Western blot analysis of total protein extracts from the infiltrated *N. benthamiana* leaves shown in Figure 2a.

Bands corresponding to the endogenous Hsp70s are indicated by open arrowhead, whereas solid arrowheads point to various Hsc70-2 fusions.

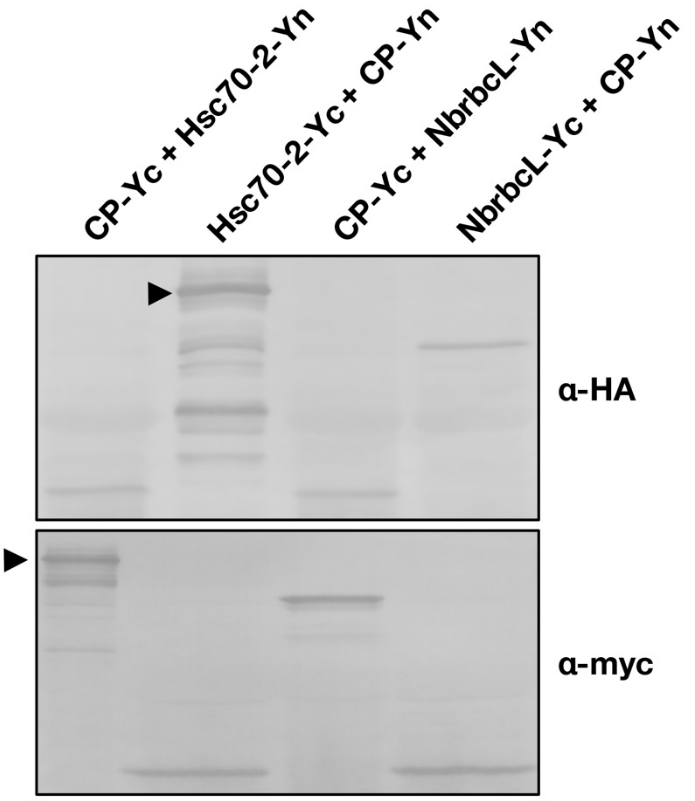

Supplementary Figure S4. Western blot analysis of total protein extracts from the infiltrated *N. benthamiana* leaves shown in Figure 2b.

Solid arrowheads indicate the Hsc70-2 fusions.

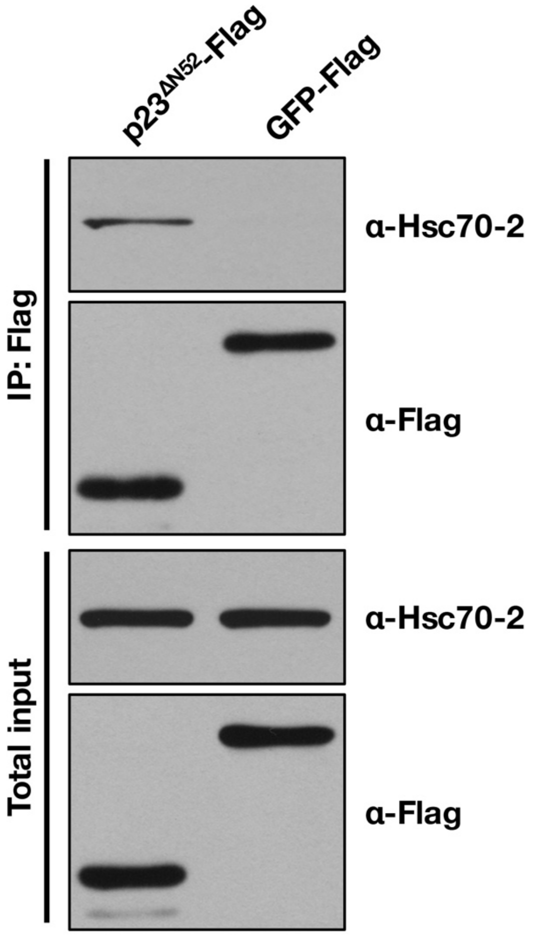

Supplementary Figure S5. Analysis of the interaction between Hsc70-2 and p23$^{\Delta N52}$ by Co-IP. GFP-Flag protein served as the negative control.

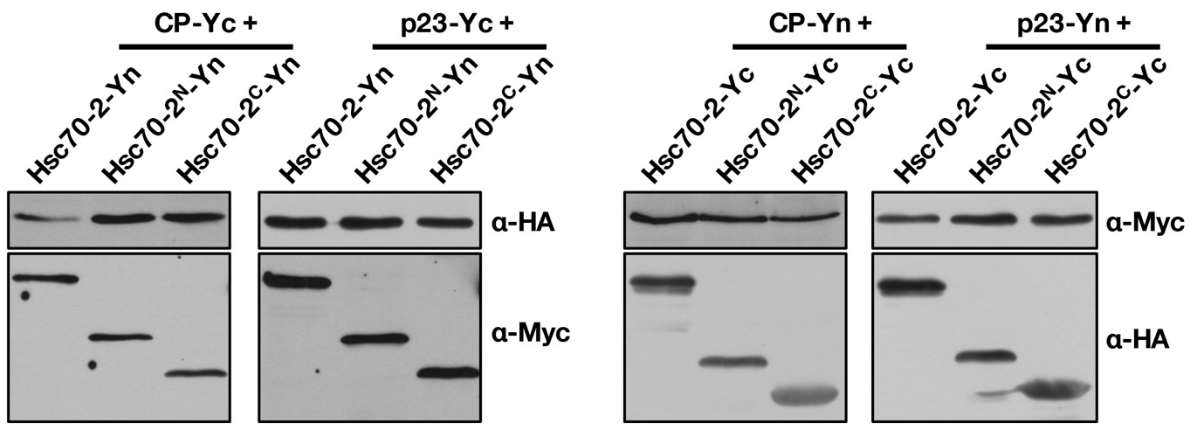

Supplementary Figure S6. Western blot analysis of total protein extracts from the infiltrated *N. benthamiana* leaves shown in Figure 3.

Various combinations of the split YFP fusions are indicated above the panels.

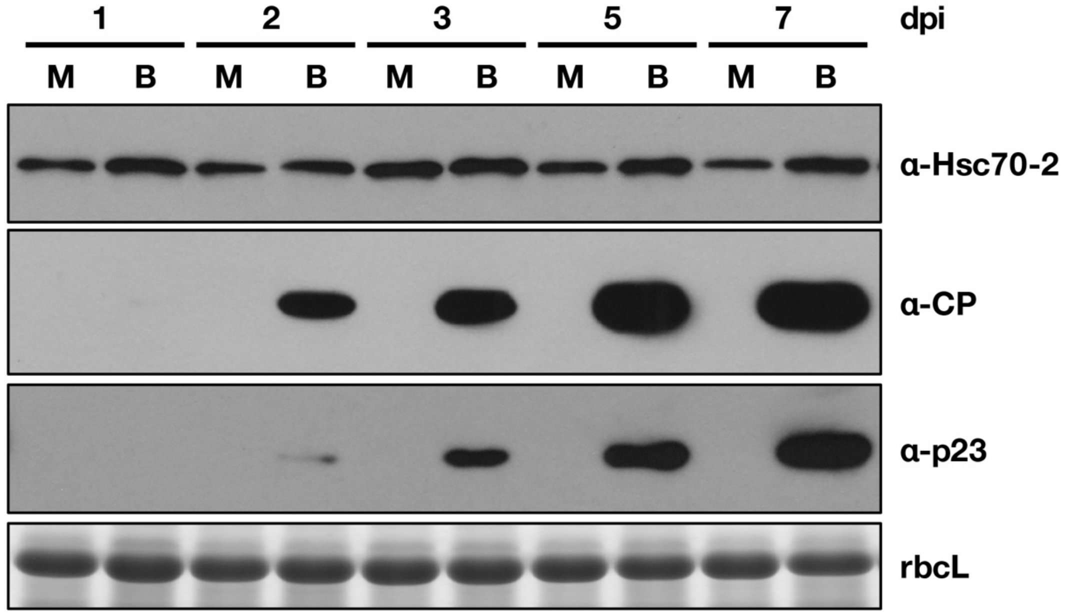

Supplementary Figure S7. Western blot analysis of Hsc70-2, CP, and p23 in mock and BBSV-inoculated *N. benthamiana* leaves at different time-points indicated above the panels.

M: buffer-treated leaves, B: BBSV-inoculated leaves. The antibodies used for Western blot analysis are indicated on the right. CBB-stained rbcL is shown as the protein loading control.

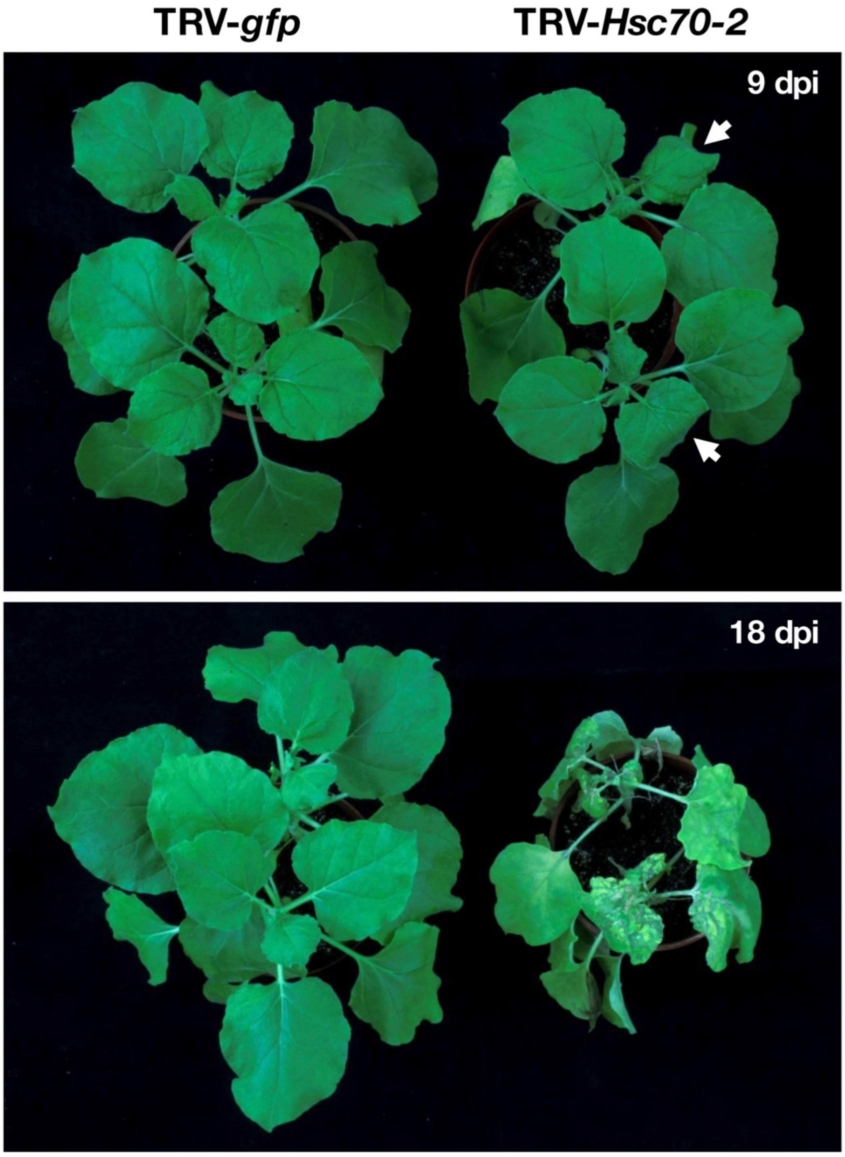

Supplementary Figure S8. Observation of the phenotypes of TRV-*gfp*- and TRV-*Hsc70-2*-inoculated *N. benthamiana* plants at 9 and 18 dpi. Arrowheads indicate the leaves showing silencing phenotype.

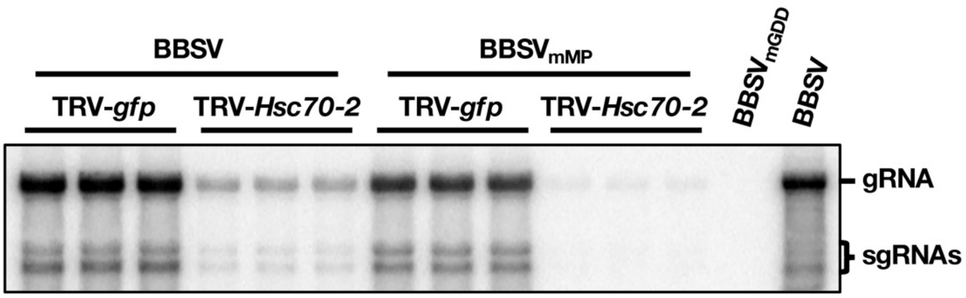

Supplementary Figure S9. Overexposure image of Northern blot results showed in Figure 5a.

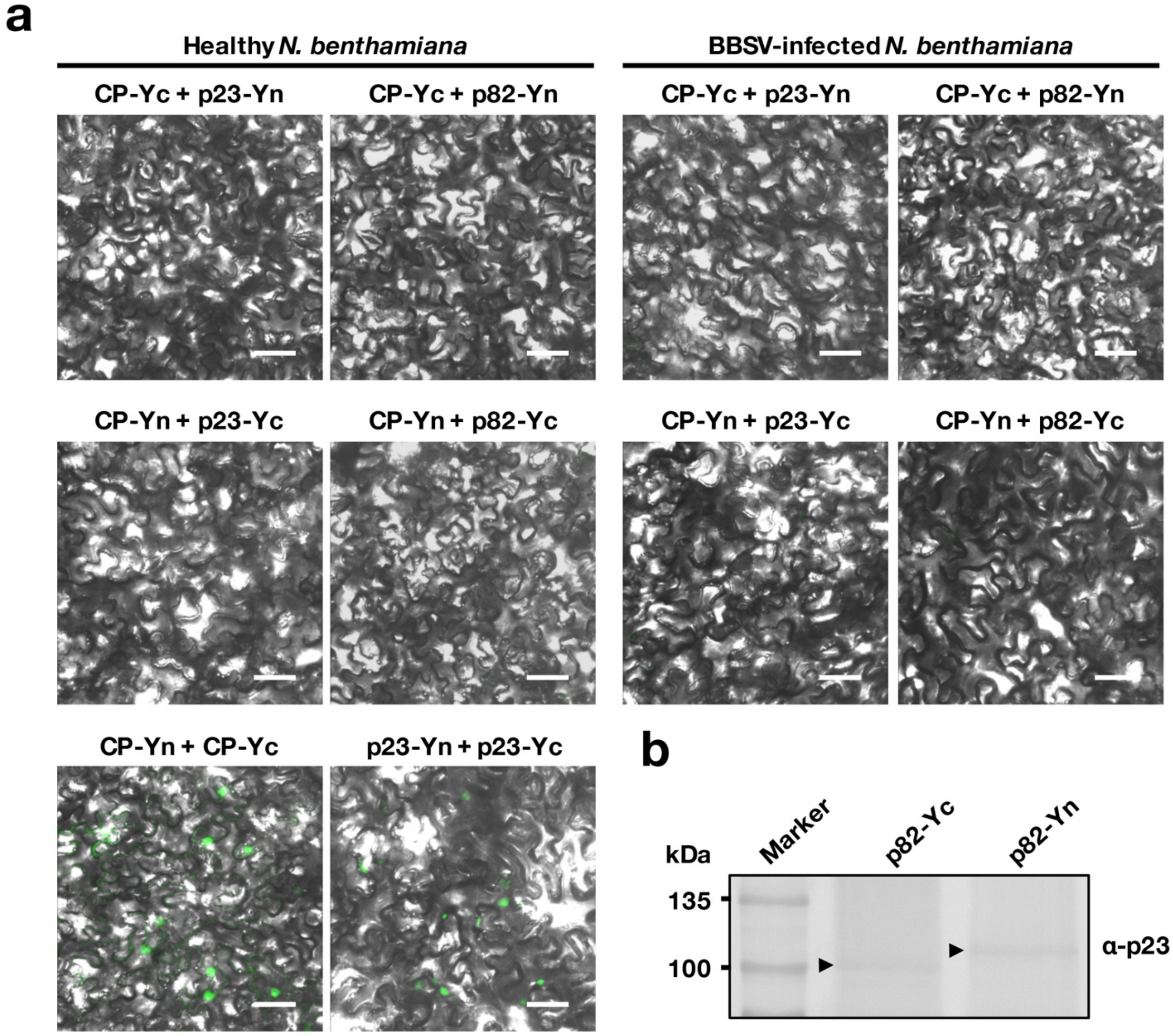

Supplementary Figure S10. CP does not interact with BBSV replication proteins.

(a) BiFC analyses of interactions between CP and p23, and between CP and p82 in healthy and BBSV-infected *N. benthamiana* leaves. Self-interactions of CP and p23 served as positive controls. Combinations of BiFC constructs are shown above each panel. Healthy and BBSV-infected plants are indicated on the top of the panels. Scale bars, 50 μm. **(b)** Western blot analyses of p82-Yn and p82-Yc proteins using antibody against p23.

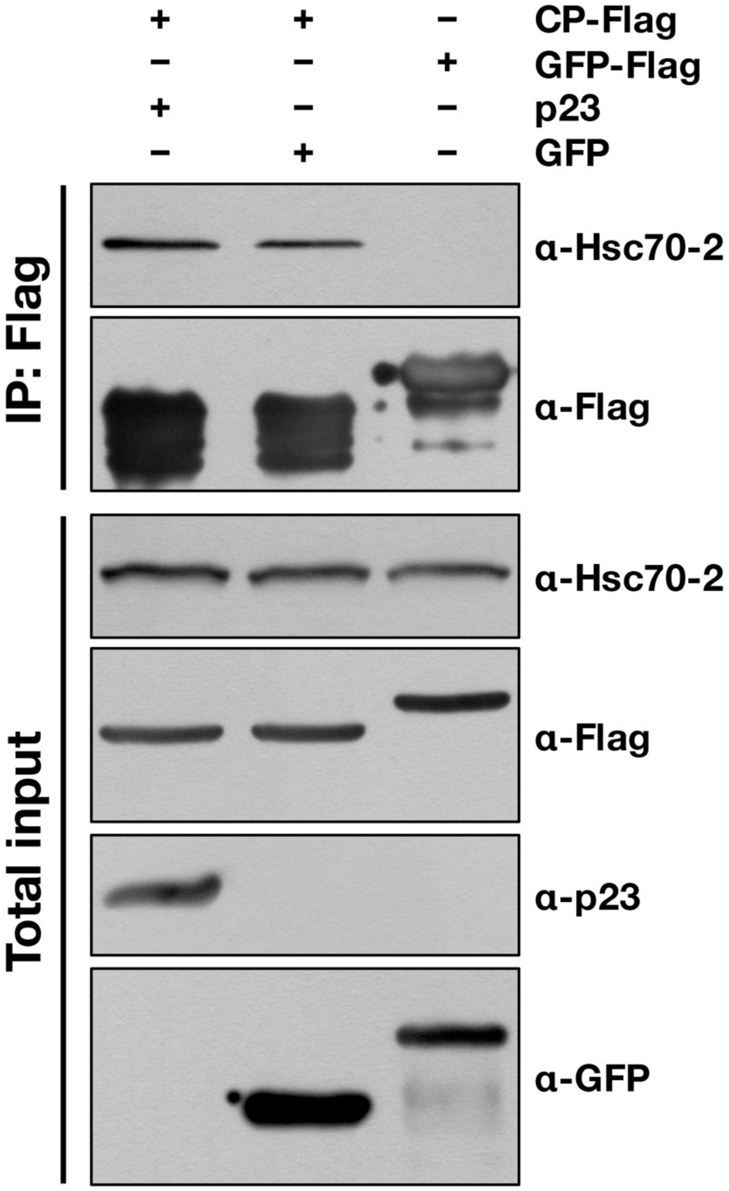

Supplementary Figure S11. p23 has no obvious effect on the interaction between Hsc70-2 and CP. *N. benthamiana* leaves were co-infiltrated with *Agrobacterium* cells carrying different constructs as shown above the panel. Co-IP analyses were conducted at 3 dpi.

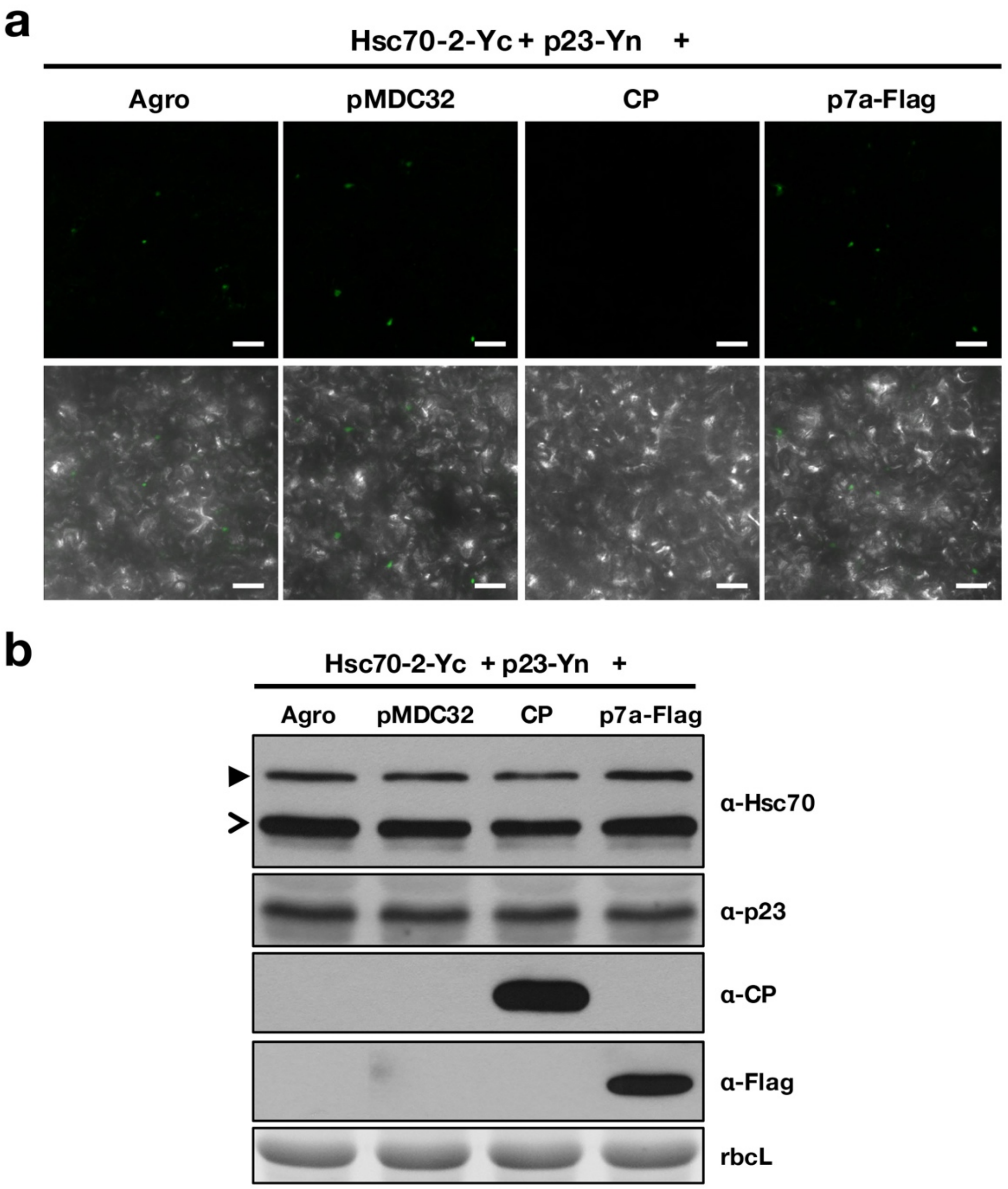

Supplementary Figure S12. p7a has no obvious influence on the interaction of Hsc70-2 with p23.

(a) BiFC analysis of the effect of CP and p7a on the interaction between Hsc70-2 and p23. *N. benthamiana* leaves co-infiltrated with *Agrobacterium* cells containing BiFC constructs and pMDC32, pMDC32-CP or pMDC32-p7a-Flag at an OD_{600} of 0.2 and 1.2, respectively. Agro indicates the *Agrobacterium* cells without carrying the plasmid. Confocal microscopic analyses were carried out at 3 dpi. Different combinations of constructs used for agroinfiltration are indicated on the top. Scale bars, 60 μm. **(b)** Western blot analysis of total protein extracts from infiltrated leaves corresponding to (a). The open arrowheads point to the bands of endogenous Hsc70-2, whereas solid arrowhead indicates the Yc-fused Hsc70-2. Antibodies used for probing the target proteins are indicated on the right side of each blot. Sample loading was assessed by CBB-stained rbcL.

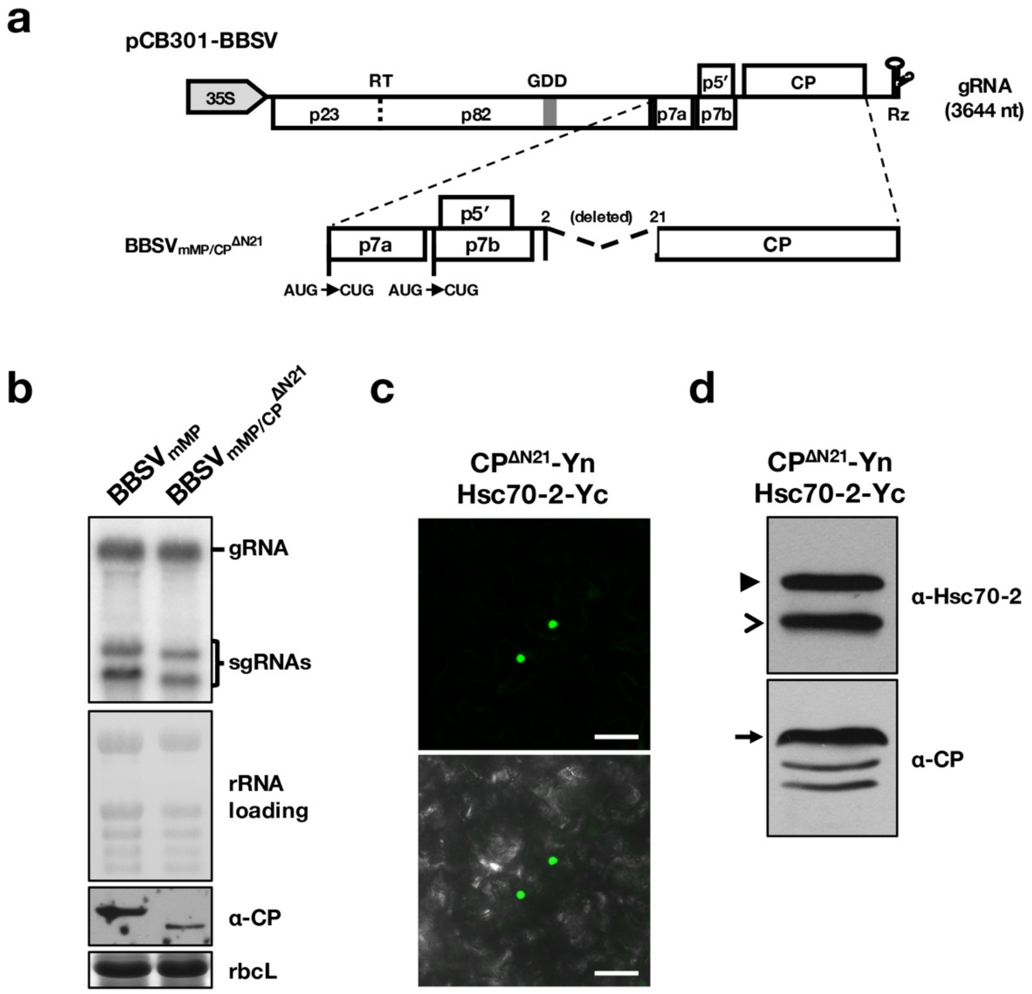

Supplementary Figure S13. Disruption of RNA binding domain within the CP has minor effect on its ability to inhibit the BBSV replication.

(a) Schematic representation of $BBSV_{mMP/CP}^{\Delta N21}$ used in *Agrobacterium tumefaciens*-mediated infection assay. **(b)** Analysis of the accumulation of the BBSV-derived mutants in *N. benthamiana* leaves. Bands corresponding to gRNA and sgRNAs as well as the antibodies used for Western blot analysis are indicated on the right. Methylene blue-stained rRNAs and CBB-stained rbcL were used as RNA and protein loading controls, respectively. **(c)** BiFC analysis of interaction between $CP^{\Delta N21}$ and Hsc70-2 in *N. benthamiana* leaves. Scale bars, 50 µm. **(d)** Western blot analysis of total protein extracts from infiltrated leaves corresponding to (c). The antibodies used for Western blot analysis are indicated on the right. Band corresponding to the endogenous Hsc70-2 is indicated by open arrowhead, while solid arrowhead points to the Yc-fused Hsc70-2. The arrow indicates the band of $CP^{\Delta N21}$-Yn.

Method used in performing the experiments shown in Supplementary Figure S2

Blue native polyacrylamide gel electrophoresis (BN-PAGE) assay

BN-PAGE analyses were performed as described previously[1] with minor modifications. Total soluble proteins extracted from *N. benthamiana* plants infected with wild-type BBSV or $BBSV_{CP\text{-}His}$ were subjected to affinity purification using Ni-NTA agarose. The purified complexes were then mixed with 40% (v/v) glycerol and separated under native conditions by BN-PAGE in a first dimension. The gel was excised and separated using denaturing SDS-PAGE followed by silver staining.

Supplementary reference

1. Swamy, M., Siegers, G. M., Minguet, S., Wollscheid, B. & Schamel, W. W. Blue native polyacrylamide gel electrophoresis (BN-PAGE) for the identification and analysis of multiprotein complexes. *Sci. STKE* **2006,** pl4 (2006).

Supplementary Table S1 Primers used in this study.

Primer	Primer sequence (5'-3') [a]	Position and description [b]	Purpose	Experiment
F-BBSV	ctctatataaggaagttcatttcatttggagaggAAGAAACCTAACCAGTTTCTCGTTG	gRNA nt 1-25	pCB301-BBSV	*Agrobacterium tumefaciens*-mediated infection assay
R-BBSV	ccgcgaggaggtggagatgccatgccgacccgggGGGCACCTGGAAGACCAGGTATATA	gRNA nt 3644-3620		
F-p7a	ACCTAACCACTTTTCCTGGAACAACAGCGTAG	gRNA nt 2213-2244	pCB301-BBSV$_{mMP}$	
R-p7a	CTACGCTGTTGTTCCAGGAAAAGTGGTTAGGT	gRNA nt 2244-2213		
F-p7b	CATTTCCACTTCTGAACTGAGCATCATTTATG	gRNA nt 2405-2436		
R-p7b	CATAAATGATGCTCAGTTCAGAAGTGGAAATG	gRNA nt 2436-2405		
F-CP	CCTAAGCGCAATAAAGGAGGCTAGTAGTCCCGCATGTCCGATGAGAC	gRNA nt 2653-2699	pCB301-BBSV$_{mMP/mCP}$	
R-CP	GTCTCATCGGACATGCGGGACTACTAGCCTCCTTTATTGCGCTTAGG	gRNA nt 2699-2653		
F-p82	GGCTTGCCAACAATGGTGCTGATTGCATGCTTGTCGTC	gRNA nt 1606-1643	pCB301-BBSV$_{mGDD}$	
R-p82	GACGACAAGCATGCAATCAGCACCATTGTTGGCAAGCC	gRNA nt 1643-1606		
F_{His}-CP	*CACCACCATCATCAT*TAGATCCCACATCCTGGTGTG	gRNA nt 3343-3363	pCPmx-7^{His}	Intermediate vector
R_{His}-CP	*GTGGCCACCTCC*ATTAATGGCAGCAGGTATTGGC	gRNA nt 3342-3321		
F_{KpnI}-p23	GGGGTACCggATGGATTCAATCCCGTATGTGATCCTGCGC	gRNA nt 36-65	pMDC32-p23-Flag<br>pMDC32-p23$^{\Delta N52}$-Flag	transient expression
F_{KpnI}-p23$^{\Delta N52}$	GGGGTACCggatgAAGATAAAGGTGGAAGTACACCCAGCCAC	gRNA nt 192-220		
R_{SpeI}-p23	GACTAGTTTTTCCATATGAGGGCCCTAGTACGGCCC	gRNA nt 644-616		
F_{KpnI}-gfp	GGGGTACCggATGGTGAGCAAGGGCGAGG	*eGFP* nb 1-19	pMDC32-GFP-Flag	
R_{SpeI}-gfp	GACTAGTCTTGTACAGCTCGTCCATG	*eGFP* nb 717-699		
F-Hsc70	ATGGCCGGAAAAGGTGAAGG	*Hsc70-2* nb 1-20	p19T-Hsc70-2	cloning vector
R-Hsc70	TTAGTCGACCTCCTCAATCTTGGG	*Hsc70-2* nb 1947-1924		
Fcz-Hsc70	gcttctgcaggggcccggggATGGACTACAAAGACCATGACGGTG	Flag nb 1-25	pSuper1300-Flag-Hsc70-2	transient expression
Rcz-Hsc70	ggatccactagtatttaaatgTTAGTCGACCTCCTCAATCTTGG	*Hsc70-2* nb 1947-1925		
F_{KpnI}-CP	GGGGTACCATGGCACCTAAGCGCAATAAAG	gRNA nt 2647-2668	pMDC32-CP<br>pCP-Flag	
R_{PacI}-CP	CCTTAATTAACTAATTAATGGCAGCAGGTATTG	gRNA nt 3345-3323		
R_{PacI}-CP^{Flag}	CCTTAATTAActa*AAGCTTGTCATCGTCATCCTTGTAGCCACCTCC*ATTAATGGCAGCAGGTATTGGC	gRNA nt 3342-3321		
F_{Flag}-Hsc70	*GATTACAAGGATGACGATGACAAGCTTGGAGGTGGC*ATGGCCGGAAAAG	*Hsc70-2* nb 1-20	p19T-Flag-Hsc70-2	Co-IP

	GTGAAGG			
R_{Flag}-Hsc70	*GATATCATGATCTTTATAATCACCGTCATGGTCTTTGTAGTCCAT*catAATCGTCGACCTGCAGGCATG	Sequence of pMD19T		
F_{DONR}-Hsc70	ggggacaagtttgtacaaaaaagcaggcttcATGGACTACAAAGACCATGAC	Flag nb 1-21	pDONR-Hsc70-2	
R_{DONR}-Hsc70	ggggaccactttgtacaagaaagctgggtcTTAGTCGACCTCCTCAATC	*Hsc70-2* nb 1947-1929	pMDC32-Flag-Hsc70-2	
F_{SmaI}-Hsc70	TCCCCCGGGaATGGCCGGAAAAGGTGAAG	*Hsc70-2* nb 1-19	pGEX-Hsc70-2	
$R_{HindIII}$-Hsc70	CCCAAGCTTTTAGTCGACCTCCTCAATCTTGG	*Hsc70-2* nb 1947-1925		
F_{MAL}-p23	atttcagaattcggatcctctAAGATAAAGGTGGAAGTACACC	gRNA nt 192-213	pMAL-p23$^{\Delta N52}$	
R_{MAL}-p23	gcttgcctgcaggtcgactCTATTTTCCATATGAGGGCC	gRNA nt 647-628		Protein expression
F_{NdeI}-gfp	GGGAATTCCATATGATGGTGAGCAAGGGCGAGG	*eGFP* nb 1-19	pET30a-GFP	
R_{SalI}-gfp	ACGCGTCGACCTTGTACAGCTCGTCCATG	*eGFP* nb 717-699		
F_{BamHI}-gfp	CGGGATCCATGGTGAGCAAGGGCGAGG	*eGFP* nb 1-19	pGEX-GFP	
$R_{HindIII}$-gfp	CCCAAGCTTTTACTTGTACAGCTCGTCCATG	*eGFP* nb 720-701		
F_{XbaI}-Hsc70	GCTCTAGAATGGCCGGAAAAGGTGAAG	*Hsc70-2* nb 1-19	pSPYCE-Hsc70-2	
R_{SpeI}-Hsc70	GGACTAGTGTCGACCTCCTCAATCTTGG	*Hsc70-2* nb 1944-1925	pSPYNE-Hsc70-2	
Fcz-Hsc70^{N}	ggatccatcgatagtactgATGGCCGGAAAAGGTGAAGG	*Hsc70-2* nb 1-20	pSPYCE-Hsc70-2^{N}	
Rcz-Hsc70^{N}	ggagcggtaccctcgaggtcCAACAAGTCTTGAACCTTCTC	*Hsc70-2* nb 1194-1174	pSPYNE-Hsc70-2^{N}	BiFC
Fcz-Hsc70^{C}	ggatccatcgatagtactgatgCTTTTGGATGTGACCCCTCTATC	*Hsc70-2* nb 1195-1217	pSPYCE-Hsc70-2^{C}	
Rcz-Hsc70^{C}	ggagcggtaccctcgaggtcGTCGACCTCCTCAATCTTGG	*Hsc70-2* nb 1944-1925	pSPYNE-Hsc70-2^{C}	
F_{XhoI}-Hsc70	CCGCTCGAGATGGCCGGAAAAGGTGAAGGTC	*Hsc70-2* nb 1-22	pTRV2-Hsc70-2	
R_{EcoRI}-Hsc70	CGGAATTCAGCAGCAAACTGCTTCTCTTC	*Hsc70-2* nb 360-340		VIGS
F_{XhoI}-GFP	CCGCTCGAGTCAAGAGTGCCATGCCCGAA	*GFP II* nb 251-270	pTRV2-gfp	
R_{BamHI}-GFP	CGCGGATCCCATCCATGCCATGTGTAATCCCA	*GFP II* nb 703-681		
F-EF1α	AGCTTTACCTCCCAAGTCATC	*EF1α* nb 979-999	RT-qPCR	
R-EF1α	AGAACGCCTGTCAATCTTGG	*EF1α* nb 1113-1094		
$F_{RT\text{-}qPCR}$-Hsc70	AAGCCACAGCAGGAGACACTCA	*Hsc70-2* nb 677-698		
$R_{RT\text{-}qPCR}$-Hsc70	TTCGCCCTCTCACATGCTGTCC	*Hsc70-2* nb 830-809		
F-nb	ATGGCACCTAAGCGCAATAAAGG	gRNA nt 2647-2669	Template of BBSV 3'-UTR-specific probes	
R-nb	GGGCACCTGGAAGACCAGGT	gRNA nt 3644-3625		

[a] Underlined letters indicate restriction enzyme sites, lowercase letters indicate sequences used for recombination or extra nucleotides introduced into the original sequences, italic letters indicate tag sequences. Letters in the rectangles denote mutated nucleotides.

[b] Numbers correspond to target nucleotide positions; a reverse order of numbers indicates that the primer is complementary to the targeted sequences.
